# Systems Analyses Reveal the Resilience of Escherichia coli Physiology during Accumulation and Export of the Nonnative Organic Acid Citramalate

**DOI:** 10.1128/mSystems.00187-19

**Published:** 2019-06-11

**Authors:** Joseph Webb, Vicki Springthorpe, Luca Rossoni, David-Paul Minde, Swen Langer, Heather Walker, Amias Alstrom-Moore, Tony Larson, Kathryn Lilley, Graham Eastham, Gill Stephens, Gavin H. Thomas, David J. Kelly, Jeffrey Green

**Affiliations:** a Molecular Biology & Biotechnology, University of Sheffield, Sheffield, United Kingdom; b Department of Biology, University of York, York, United Kingdom; c Bioprocess, Environmental and Chemical Technologies, University of Nottingham, Nottingham, United Kingdom; d Cambridge Centre for Proteomics, Department of Biochemistry, University of Cambridge, Cambridge, United Kingdom; e biOMICS Mass Spectrometry Facility, Department of Animal and Plant Sciences, University of Sheffield, Sheffield, United Kingdom; f Lucite International, Wilton, United Kingdom; Northwestern University

**Keywords:** *Escherichia coli*, bioproduction of chemicals, citramalate, citramalic acid, fed-batch fermentation, lipidomics, metabolomics, proteomics, transcriptomics

## Abstract

Productivity of bacterial cell factories is frequently compromised by stresses imposed by recombinant protein synthesis and carbon-to-product conversion, but little is known about these bioprocesses at a systems level. Production of the unnatural metabolite citramalate in Escherichia coli requires the expression of a single gene coding for citramalate synthase. Multiomic analyses of a fermentation producing 25  g liter^−1^ citramalate were undertaken to uncover the reasons for its productivity. Metabolite, transcript, protein, and lipid profiles of high-cell-density, fed-batch fermentations of E. coli expressing either citramalate synthase or an inactivated enzyme were similar. Both fermentations showed downregulation of flagellar genes and upregulation of chaperones IbpA and IbpB, indicating that these responses were due to recombinant protein synthesis and not citramalate production. Citramalate production did not perturb metabolite pools, except for an increased intracellular pyruvate pool. Gene expression changes in response to citramalate were limited; none of the general stress response regulons were activated. Modeling of transcription factor activities suggested that citramalate invoked a GadW-mediated acid response, and changes in GadY and RprA regulatory small RNA (sRNA) expression supported this. Although changes in membrane lipid composition were observed, none were unique to citramalate production. This systems analysis of the citramalate fermentation shows that E. coli has capacity to readily adjust to the redirection of resources toward recombinant protein and citramalate production, suggesting that it is an excellent chassis choice for manufacturing organic acids.

**IMPORTANCE** Citramalate is an attractive biotechnology target because it is a precursor of methylmethacrylate, which is used to manufacture Perspex and other high-value products. Engineered E. coli strains are able to produce high titers of citramalate, despite having to express a foreign enzyme and tolerate the presence of a nonnative biochemical. A systems analysis of the citramalate fermentation was undertaken to uncover the reasons underpinning its productivity. This showed that E. coli readily adjusts to the redirection of metabolic resources toward recombinant protein and citramalate production and suggests that E. coli is an excellent chassis for manufacturing similar small, polar, foreign molecules.

## Abstract (precis)

Citramalate is an attractive biotechnology target because it is a precursor of methylmethacrylate, which is used to manufacture Perspex and other high-value products. Engineered E. coli strains are able to produce high titers of citramalate, despite having to express a foreign enzyme and tolerate the presence of a nonnative biochemical. A systems analysis of the citramalate fermentation was undertaken to uncover the reasons underpinning its productivity. This showed that E. coli readily adjusts to the redirection of metabolic resources toward recombinant protein and citramalate production and suggests that E. coli is an excellent chassis for manufacturing similar small, polar, foreign molecules.

## INTRODUCTION

Citramalic acid can be used as a chemical precursor to methylmethacrylate (MMA [1, 2]), a relatively high-value bulk chemical with a wide range of applications, including the manufacture of Perspex. High-titer bioproduction of citramalate to >82 g liter^−1^ has been developed simply by expressing an evolved Methanococcus jannaschii citramalate synthase (CimA3.7 [3]) in Escherichia coli to catalyze the reaction of pyruvate and acetyl coenzyme A (acetyl-CoA) to produce citramalate (4, 5). Although some minor by-products can be formed in the fed-batch fermentation process, these can be converted to MAA alongside the citramalate in the chemical conversion process (2, 5). Further work to eliminate their formation using chassis gene deletions (*ackA-pta*, *aceB*, *glcB*, *gltA*, *leuCD*, and *poxB*) resulted in much lower yields of citramalate (6–8) and increased process costs (5). This prior work has demonstrated the potential to develop bio-based production of citramalate in a simple fed-batch process using a minimally engineered E. coli strain as a precursor for manufacturing MMA.

The product titers are surprising given that citramalate is not formed naturally by E. coli and that citramalate formation drains two central metabolites—pyruvate and acetyl-CoA. Therefore, time-resolved multiomic analyses (transcriptome, proteome, metabolome, and lipidome) of the citramalate fermentation were undertaken to understand the effects of both recombinant protein synthesis (CimA3.7) and citramalate production on the host E. coli chassis. Citramalate production had relatively few effects on the host transcriptome, proteome, lipidome, or metabolome, providing a simple explanation for the exceptional product titers and yields.

## RESULTS AND DISCUSSION

### Citramalate production and multiomic sampling regime in a high-cell-density, fed-batch fermentation.

**FIG 1:**
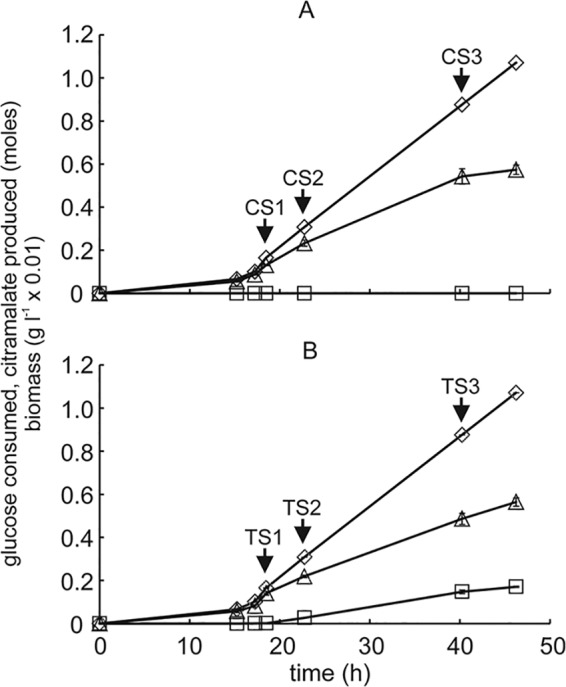
Fed-batch cultures of E. coli expressing inactivated (A) and active (B) CimA3.7. Biomass (triangles), glucose consumed (diamonds), citramalate produced (squares), and sampling points (C indicates control, inactivated CimA3.7_dead_; T indicates test, active CimA3.7) for omic analyses are indicated. Sample points CS1 and TS1 are immediately before induction of inactive CimA3.7_dead_ protein or active CimA3.7, respectively, with l*-*arabinose.

Escherichia coli expressing citramalate synthase (CimA3.7) or an inactivated variant (CimA3.7_dead_) was grown in triplicate, high-cell-density, glucose-limited fed-batch cultures under aerobic conditions. Preliminary batch culture experiments showed that, after induction with arabinose, CimA3.7_dead_ was produced to similar levels (∼4% of total protein) to CimA3.7 and that CimA3.7_dead_ lacked catalytic activity (see Fig. S1 in the supplemental material). The triplicate control and test fermentations were reproducible, such that the data could be combined (Fig. 1). After initial batch growth for 18.5 h, the bioprocess continued under a fed-batch regimen, and synthesis of inactive or active CimA3.7 was induced with l-arabinose at an optical density at 600 nm (OD_600_) of ∼50. The subsequent feeding regimen was implemented to produce citramalate concentrations close to the 50% effective concentration (EC_50_ [173 ± 7 mM]) (5). For subsequent analyses, samples were obtained before induction (S1) and then 4 h (S2) and 21.5 h (S3) postinduction for triplicate control (C [CimA3.7_dead_]) and test (T [CimA3.7]) fermentations (Fig. 1).

10.1128/mSystems.00187-19.1 FIG S1 The CimA3.7 variant (His192Ala; CimA3.7_dead_) lacks citramalate synthase activity but is expressed to similar levels to wild-type CimA3.7 in E. coli BW25113. (A) The citramalate synthase activities of crude cell-free extracts of E. coli transformed with pBAD24-*cimA3.7 (*CimA3.7, squares) or pBAD24-*cimA3.7_dead_* (CimA3.7_dead_, triangles) were measured. Data are means and standard deviations for triplicate assays. (B) Coomassie blue-stained denaturing polyacrylamide gel of whole-cell extracts from batch cultures of E. coli BW25113 expressing CimA3.7 or CimA3.7_dead_. Cultures were grown in LB and induced with l*-*arabinose (0.02%). Ten micrograms of total protein was loaded on SDS-polyacrylamide gels, which were stained with Coomassie blue after electrophoresis. The locations of CimA3.7 and CimA3.7_dead_ are indicated (arrow). Densitometry using Image J suggested that CimA3.7 and CimA3.7_dead_ accumulate to similar levels (4% and 3.5% of total protein, respectively) (58). Download FIG S1, TIF file, 1.1 MB. Copyright © 2019 Webb et al. 2019 Webb et al. This content is distributed under the terms of the Creative Commons Attribution 4.0 International license.

Both control and test fermentations consumed all the glucose provided in the culture feed, with higher biomass production in the control compared to the test (conversion efficiencies for biomass with glucose of 0.30 ± 0.01 g cell dry weight [cdw] g glucose^−1^ for the controls and 0.26 ± 0.01 g cdw g glucose^−1^ for the test fermentations [Fig. 1]). As expected, the major difference was the accumulation of citramalate to 153 ± 5 mM in the test fermentations, which equates to 0.129 ± 0.003 mol citramalate per mol glucose consumed (Fig. 1B). After induction, the control fermentations produced low concentrations of formate (∼3 mM) and acetate (maximally ∼1.7 mM). Acetate, but not formate, was also detected (maximally ∼1.1 mM) in the test fermentations.

Proton nuclear magnetic resonance (NMR) of culture supernatants confirmed that citramalate was the dominant exo-metabolite in the test fermentations, along with ∼30-fold-lower concentrations of citraconate (see Fig. S2 in the supplemental material). The overall carbon recoveries (assuming that 1 g of biomass corresponds to the production of 1 g of CO_2_) were 85% ± 2%, 81% ± 5%, and 67% ± 4% for control sample 1 [CS1], CS2, and CS3, respectively, and 91% ± 6%, 83% ± 2%, and 73% ± 3% for test sample 1 [TS1], TS2, and TS3, respectively (Fig. 1). A multiomic approach was taken to enhance understanding of the mechanisms underpinning adaptation to recombinant protein/product accumulation in high-cell density fermentations at a system level.

10.1128/mSystems.00187-19.2 FIG S2 Citramalate is the dominant exometabolite in cultures of E. coli expressing CimA3.7. Shown are proton NMR spectra of culture supernatants taken from control (A) and test (B) fermentations at the indicated sampling points. Chemical shifts assigned to citramalate based upon an authentic citramalate standard (Sigma-Aldrich [1.34, 2.24, to 2.77 ppm]) are indicated. (C) An enlarged image of overlayed TS2 (red) and CS2 (black) spectra showing peaks assigned to citraconate based upon an authentic citraconate standard (Sigma-Aldrich [1.91 and 5.6 ppm]) in TS2. Download FIG S2, TIF file, 2.6 MB. Copyright © 2019 Webb et al. 2019 Webb et al. This content is distributed under the terms of the Creative Commons Attribution 4.0 International license.

To investigate cellular adaptations to production of citramalate, samples of the citramalate production fermentation were taken at representative time points before and after induction of CimA3.7 or CimA3.7_dead_. Samples were used for comprehensive multiomic analyses of mRNA, protein, lipid and metabolite levels.

### Citramalate production results in an increased intracellular pyruvate pool.

**FIG 2:**
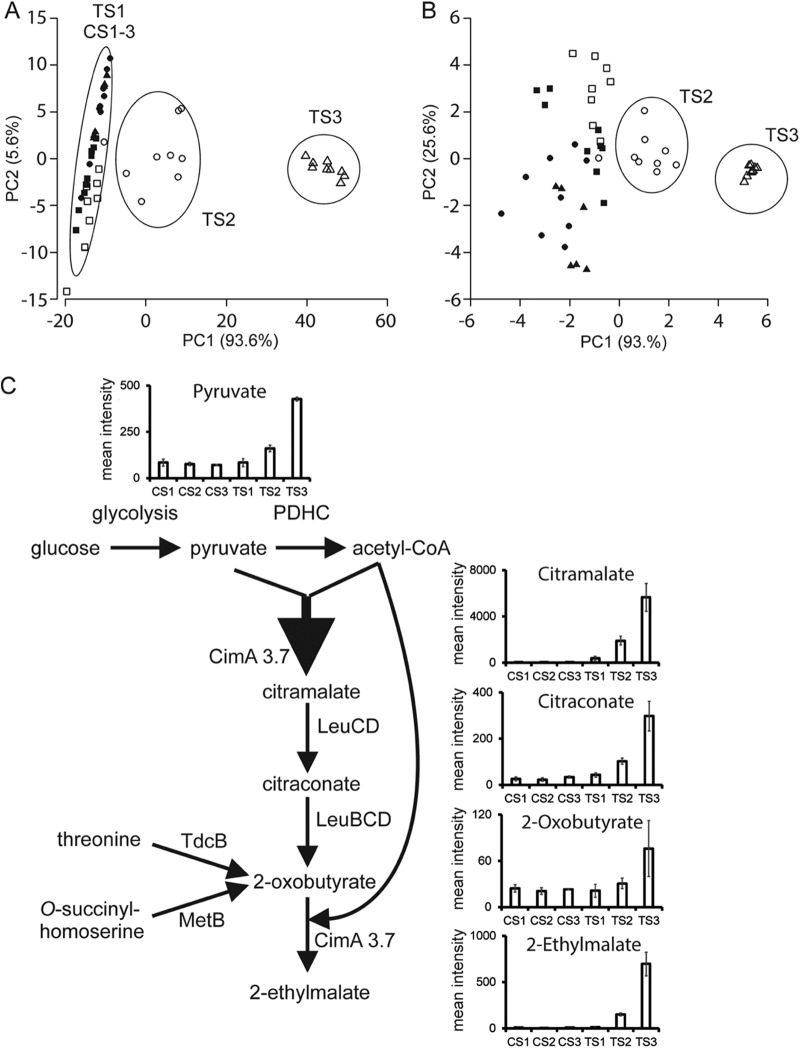
Perturbation of chassis metabolites during production of citramalate. (A) Principal-component analysis (PCA) score plots showing the first and second principal components of the endo-metabolome for CS1 to 3 (filled squares, circles, and triangles) and TS1 to 3 (open squares, circles, and triangles). The analysis is of three independent biological replicates. (B) Reanalysis of the endo-metabolome without citramalate and phosphate. (C) Pyruvate generated from the glucose feed by glycolysis is converted to acetyl-CoA and CO_2_ by the pyruvate dehydrogenase complex (PDHC). ESI-TOF MS data of the endo-metabolome suggested that citramalate is efficiently produced and accumulates in the cytoplasm (maximum mean intensity of ∼6,000) by the action of CimA3.7 on pyruvate and acetyl-CoA, regenerating CoASH. The accumulated citramalate is mostly excreted (∼150 mM in the culture medium), but some is converted to citraconate (maximum mean intensity of ∼300), catalyzed by isopropylmalate isomerase (LeuCD), and is detected in both the endo- and exo-metabolome (∼3 mM in the culture medium) at low levels. Citraconate can be converted to 2-oxobutyrate (maximum mean intensity of ∼50) by the combined action of LeuBCD. 2-Oxobutyrate can also be synthesized by the action of threonine dehydratase or *O*-succinylhomoserine lyase (MetB). 2-Oxobutyrate is a substrate for CimA3.7, yielding 2-ethylmalate (maximum mean intensity of ∼800).

Before induction of CimA, the control and test fermentation endo-metabolomes were similar, clustering together in principal-component analysis (PCA) plots (Fig. 2). After induction, the endo-metabolomes of the control and test fermentations were clearly differentiated by citramalate (bin mass of 148.08 Da) (Fig. 2A). Reanalysis of these data after removal of the citramalate and phosphate mass bins revealed further differences between the control and test fermentations after induction (Fig. 2B). These included bin masses of 88.05 Da (identified as pyruvate by tandem mass spectrometry [MS-MS]), 130.08 Da (citraconic acid), 149.08 Da (possibly methionine), and 162.08 Da (2-ethylmalate). Citraconic acid is formed from citramalate by the E. coli 3-isopropylmalate dehydratase LeuCD (Fig. 2C) (3). However, previous attempts to remove this activity via individual *leuC* and *leuD* mutations increased fermentation cost and complexity through the requirement to feed leucine and did not enhance citramalate production in shake flask cultures (7). In an industrial process, citraconate is not problematic since it can be converted to methacrylic acid using the same process as that developed for citramalate (4, 5). However, the formation of 2-ethylmalate was unexpected, but resolved by finding novel activity of CimA3.7 when 2-oxobutyrate was supplied in place of the cognate substrate, pyruvate, *in vitro* (see Fig. S3 in the supplemental material). Hence, 2-ethylmalate was formed by CimA3.7-catalyzed condensation of acetyl-CoA with 2-oxobutyrate (Fig. 2C). 2-Oxobutyrate can be formed via the citramalate pathway catalyzed by CimA and LeuBCD or in reactions catalyzed by threonine deaminase and *O*-succinylhomoserine lyase (Fig. 2C). Although relatively low, the intracellular concentration of 2-ethylmalate was ∼1.7-fold greater than the concentration of pyruvate. Production of 2-ethylmalate represents an additional drain on acetyl-CoA pools, reducing the availability of acetyl-CoA for citramalate production. Possible interventions to limit production of the 2-ethylmalate by-product would be deletion of 3-isopropylmalate dehydratase, threonine deaminase, and *O*-succinylhomoserine lyase activities or improve the substrate specificity of CimA3.7. However, it should be noted that 2-ethylmalate is not detectable in the culture supernatant and would not, therefore, affect the purity of the citramalate product. Furthermore, elimination of these 2-ethylmalate-forming reactions would require expensive nutritional supplementation to compensate for the ensuing auxotrophy (5). Therefore, further metabolic engineering to eliminate 2-ethylmalate would not provide any benefits.

10.1128/mSystems.00187-19.3 FIG S3 Citramalate synthase (CimA3.7) exhibits low but significant activity with 2-oxobutyrate. Reombinant CimA3.7 was expressed in E. coli, and activity with either pyruvate (squares) or 2-oxobutyrate (triangles), in the presence of acetyl-CoA, was measured. As a control, extracts from cultures of E. coli transformed with the vector (pBAD24, circles) were also tested for activity. The data are means and standard deviations from triplicate assays. Download FIG S3, TIF file, 0.6 MB. Copyright © 2019 Webb et al. 2019 Webb et al. This content is distributed under the terms of the Creative Commons Attribution 4.0 International license.

The clear differences between the endo-metabolome of the test fermentations postinduction compared to the controls suggested that there are changes that extend beyond simply redirecting pyruvate and acetyl-CoA away from aerobic respiratory metabolism and biomass production toward citramalate synthesis, including the intracellular production of 2-ethylmalate. Therefore, changes in the chassis transcriptome, proteome, and lipidome during control and test fermentations were analyzed for responses to the metabolic burdens imposed by heterologous protein synthesis and citramalate production.

### Expression of recombinant citramalate synthase affects gene expression more than the production of citramalate.

The transcriptional response to CimA3.7 and citramalate production was analyzed. In the control fermentation, 303 genes exhibited altered transcription (≥2-fold, adjusted *P* value of ≤0.05), 4 h after induction of CimA3.7_dead_ synthesis and 36 genes after 21.5 h, representing a total of 316 genes (see Table S1A and Fig. S4 in the supplemental material). For the control fermentations, only genes linked to flagellar biosynthesis were identified as being significantly enriched (GO:0071978; 7.79, *P* = 0.024, Fisher’s exact test with false-discovery rate [FDR] multiple-test correction) by the AmiGO gene ontology software (9). For the test fermentations, only 15 genes met the significance criteria 4 h after induction and 42 genes 21.5 h postinduction, representing a total of 53 different genes, of which 39 were also differentially regulated in the control (Table S1B). As observed for the control fermentations, flagellar genes were significantly enriched (GO:0071978; 29.02, *P* = 0.0007) in the test fermentations, suggesting that downregulation of flagellar biosynthesis is an adaptation to high-cell density recombinant protein production. Downregulation of flagellar synthesis has been observed during recombinant protein synthesis in other E. coli strains, and it has been suggested that chassis performance might be enhanced by shutting down the flagellar machinery (10–13).

10.1128/mSystems.00187-19.4 FIG S4 Volcano plots showing the changes in transcriptome and proteome between the production fermentation and control fermentation. (A) Transcriptome T2, (B) transcriptome T3, (C) proteome T2, (D) proteome T3. Black dots are proteins or genes exhibiting a fold change of >2 and a *P* value of <0.05. Red dots are proteins or genes exhibiting a fold change of >2 and a false-discovery rate adjusted *P* value of <0.05. Download FIG S4, TIF file, 0.5 MB. Copyright © 2019 Webb et al. 2019 Webb et al. This content is distributed under the terms of the Creative Commons Attribution 4.0 International license.

10.1128/mSystems.00187-19.6 TABLE S1 (A) Transcripts exhibiting significant changes after induction of inactive CimA3.7A (control fermentations). (B) Transcripts exhibiting significant changes after induction of active CimA3.7 (test fermentations). (C) Transcripts exhibiting significant changes in the test fermentations relative to the control fermentations. (D) Proteins exhibiting significant changes after induction of inactive CimA3.7A (control fermentations). (E) Proteins exhibiting significant changes after induction of active CimA3.7 (test fermentations). (F) Proteins exhibiting significant changes in the test fermentations relative to the control fermentations. Download Table S1, XLSX file, 0.03 MB. Copyright © 2019 Webb et al. 2019 Webb et al. This content is distributed under the terms of the Creative Commons Attribution 4.0 International license.

Genes that were only upregulated in the test fermentations were deemed to be potentially specific responses to citramalate production (Table 1). These included two efflux pumps (*alaE* and *mdtE*) and components of the E. coli acid response (*gadC* and *hdeB*). This suggested that citramalate production was associated with acid stress and that AlaE and/or MdtE might contribute to efflux of citramalate from the cytoplasm.

**TABLE 1:** Differentially regulated operons after induction of test fermentations expressing active CimA3.7 and producing citramalate^a^

Gene or operon	Function	Maximum fold change	Direction of regulation
*mdtEF-tolC*	Multidrug resistance efflux transporter	4.6	Up
*malEFG*	Maltose transport	3.1	Down
*gadB****C***	Acid resistance	2.3	Up
*treBC*	EnzII_Tre_ PTS; trehalase 6-P hydrolase	2.3	Down
*entC****A****BEH*	Enterobactin synthesis	2.3	Up
*hdeA****B****-yhiD*	Acid resistance	2.1	Up
*alaE*	l-Alanine export	2.0	Up

a Fold change is for the first gene in the operon unless indicated otherwise by boldface type.

The global gene expression data described above suggested that the transcriptional response to citramalate synthesis was limited. To complement that analysis, the TFInfer software was used to infer any changes in the activities of 201 regulators during the control and test fermentations (14). The statistical inference of changes in the activities of transcription factors has the potential to extract hidden information in the transcriptomic data by revealing the regulators (and by extension their cognate signals) responsible for the observed changes in transcript abundance. This probabilistic approach requires a connectivity matrix linking transcription factors to transcription units, and this was constructed using the list of E. coli transcription factors and the operons that they regulate in RegulonDB (15).

**FIG 3:**
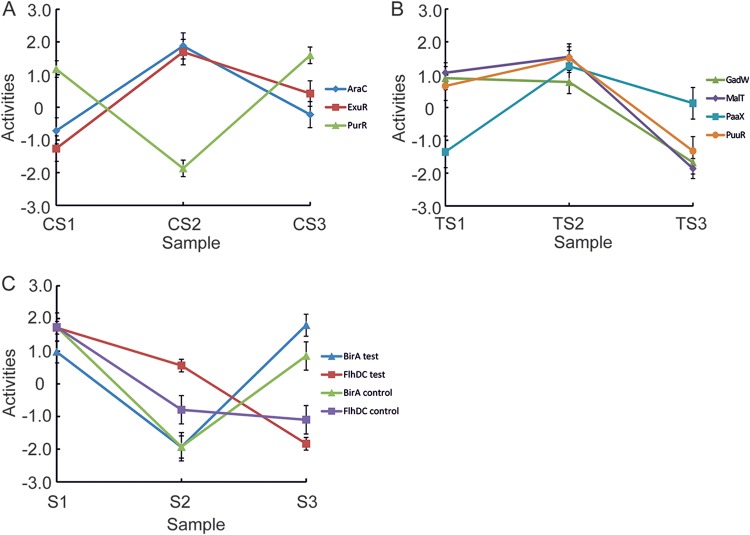
Changes in transcription factor activities inferred from transcriptomic data. The TFInfer software (14) was used to identify transcription factors that exhibited a significant change in activity (signal/noise ratio of ≥4) upon induction of control and test fermentations. Induction of the control fermentations resulted in the production of Cim3.7_dead_; induction of test fermentations caused the synthesis of CimA3.7 and the consequent accumulation of citramalate. The cultures were sampled before (S1) and 4 (S2) and 21.5 (S3) h after induction. (A) Transcription factors that only met the significance criteria in the control fermentations. (B) Transcription factors that only met the significance criteria in the test fermentations. (C) Transcription factors that exhibited altered activity after induction in both the control and the test fermentations. Transcription factors can be identified by reference to the keys provided in each panel. The error bars are the standard deviation provided by the posterior distribution.

The transcription factors exhibiting the greatest changes in activity (signal/noise ratio of ≥4) in the control fermentations were AraC, BirA, ExuR, FlhDC, and PurR (Fig. 3A and C; see Table S2 in the supplemental material). For the test fermentations, five transcription factors, including BirA and FlhDC (BirA, FlhDC, GadW, MalT, PaaX, and PuuR), responded with a signal-to-noise ratio of ≥4 (Fig. 3B and C; Table S2). The TFInfer software also reveals the target genes that are most heavily influenced by each transcription factor (Table S2). These analyses further confirm that inactivation of FlhDC and the consequent inhibition of flagellar gene expression are a core adaptation to recombinant protein production and that citramalate production initiates a GadW-mediated acid response and modulation of MalT-regulated sugar uptake. The latter is notable because enhanced succinate production by E. coli strain HX024, an evolved derivative of strain Suc-T110, was associated with increased expression of the *malK*-*lamB*-*malM* operon (16). Thus, changes in MalT-mediated regulation might be a common response to the production of organic acids using an E. coli chassis. Nevertheless, none of the major stress-responsive transcription factors (e.g., cyclic AMP receptor protein [CRP], glucose starvation; fumarate and nitrate reductase [FNR], oxygen starvation; OxyR/SoxRS, oxidative stress; CpxAR, envelope stress; CspA, cold shock; EvgSA/PhoPQ/GadE/GadX/EnvZ-OmpR, low pH; NhaR, sodium stress; LexA, DNA damage; CusR/CueR/Fur/Zur/ZntR, metal ion homeostasis) were significantly activated in response to CimA3.7_dead_/CimA3.7 and/or citramalate production.

10.1128/mSystems.00187-19.7 TABLE S2 Gene most influenced by transcription factors in the test and control fermentations. Download Table S2, XLSX file, 0.01 MB. Copyright © 2019 Webb et al. 2019 Webb et al. This content is distributed under the terms of the Creative Commons Attribution 4.0 International license.

An alternative analytical approach is to calculate the differences in transcript abundance between the samples CS2 and TS2 and CS3 and TS3 after normalization to the respective preinduction samples CS1 and TS1 (Fig. 1). The data were then filtered to include transcripts exhibiting differences in abundance of ≥2-fold (adjusted *P* value of ≤0.05) in the test samples compared to the control (Table S1C). These criteria were met by only seven genes: two associated with sulfur metabolism (*cysD* and *cysW*), two with transporter functions (*exuT* and *malK-lamB-malM*), one coding for a hypothetical protein (*ygbK*), and genes associated with flagellar (*fliA*) and tryptophan (*trpD*) biosynthesis (Table 2). Despite the diversion of pyruvate and acetyl-CoA away from central metabolism toward citramalate production and the potential for citramalate-induced osmotic stress in the postinduction production cultures, extensive changes in gene expression were not observed (Table S1C).

**TABLE 2:** Operons associated with production of citramalic acid by an E. coli cell factory that were differentially regulated ≥2-fold (adjusted *P* value of ≤0.05) relative to control fermentations^a^

Gene or operon	Function	Fold change	Direction of regulation
*trpLE****D****CBA*	Tryptophan biosynthesis	5.1	Up
*ygbJ****K***	Hypothetical protein	4.1	Down
*malK-lamB-malM*	Maltose transport	3.8	Up
*exuT*	Hexuronate transport	3.6	Down
*cysDNC*	Sulfur metabolism	3.1	Up
*cysPU****W****AM*	Sulfur metabolism	2.8	Up
*fliAZ-tcyJ*	Flagellar biosynthesis	2.6	Up

a Fold change is for the first gene in the operon unless indicated otherwise by boldface type.

### Roles of sRNAs during citramalate production.

**FIG 4:**
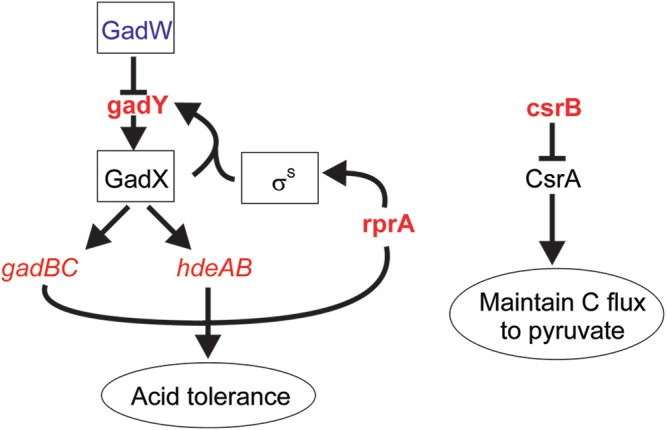
Responses of the E. coli chassis to production of citramalate. Inference of changes in transcription factor activities suggested that the activity of GadW decreased upon synthesis of citramalate (Fig. 3B). Accordingly, the abundance of the GadW-repressed sRNA GadY increased (Table 3). GadY acts to stabilize the mRNA encoding GadX, which along with GadE activates expression of the *gadBC* and *hdeAB* operons (coding for the glutamate decarboxylase-glutamate:4-aminobutyrate antiporter acid tolerance system and the periplasmic chaperones [Table 1]). Citramalate synthesis was also associated with enhanced abundance of the RprA sRNA, which enhances σ^S^-dependent acid tolerance by increasing translation of RpoS (Table 3). The redirection of carbon from biomass to citramalate production resulted in decreased abundance of the sRNA CsrB, which is an antagonist of the translational regulator CsrA (Table 3). Higher CsrA activity in the production strain maintains carbon flux toward pyruvate, rather than to storage as glycogen, thereby enhancing citramalate productivity. Transcription factors are boxed, sRNAs are in bold, operons are italic, processes are circled, increased abundance is red, and lower abundance is blue.

As well as permitting changes in the expression of mRNAs to be detected, the microarray design included 132 custom probes to report on the abundances of 77 noncoding RNAs. The abundances of 13 small regulatory RNAs (sRNAs) changed significantly upon induction of CimA3.7 (Table 3). Three of these sRNAs (ChiX, RyhB, and RyjA) were regulated similarly in the control and citramalate production fermentations. Increased abundance of ChiX results in inhibition of chitosugar uptake and utilization, enhanced levels of RyhB are associated with iron starvation and lowered synthesis of iron-containing proteins, and RyjA (also known as SarL) inhibits the synthesis of trigger factor (a ribosome associated protein-folding chaperone) in response to lower protein synthesis in stationary phase (17). Nine sRNAs were significantly regulated in the control fermentations, but not in the test fermentations, postinduction, but these sRNAs were not significantly differentially regulated when their abundances in the test fermentations were compared to those in the controls (Table 3). The 10th sRNA, CsrB, exhibited opposite regulation in the control and production fermentations, being upregulated in the former and downregulated in the latter postinduction. The enhanced levels of CsrB should decrease the activity of CsrA, which acts as a repressor of gene expression in stationary phase, consistent with the higher growth rate/yield of the control fermentations (18) (Fig. 4). More specifically, CsrA maintains carbon flux toward pyruvate, rather than to storage as glycogen. Therefore, the decreased abundance of CsrB may increase glycolytic flux beyond the capacity of the pyruvate dehydrogenase complex. This increase in glycolytic flux would contribute to the observed increase in intracellular pyruvate. An increase in glycolytic flux might be triggered by a decreased capacity to generate ATP by oxidative phosphorylation created by the diversion of pyruvate and acetyl-CoA away from the citric acid cycle toward citramalate production. This diversion likely constrains the ability to generate reducing equivalents via the tricarboxylic acid (TCA) cycle and subsequently ATP by oxidative phosphorylation. Under such circumstances, lower ATP availability might invoke increased glycolytic flux, as observed when ATP concentrations are manipulated by expression of an uncoupled ATPase (19).

**TABLE 3:** Differentially regulated sRNA molecules during citramalate production

sRNA	Function	Fold change					
		Preinduction samples^*a*^				Production strain vs control strain^*b*^	
		CS2	CS3	TS2	TS3	TS2:CS3	TS3:CS3
ChiX	Chitosugar catabolism	3.7	1.7	2.5	1.7	NS^*c*^	NS
CsrB	Antagonist of global translational regulator CsrA	3.0	1.3	NS	0.3	0.4	0.2
GadY	Acid tolerance—enhances stability of *gadX* transcript	NS	NS	1.3	2.1	2.0	3.0
MicF	Regulation of OmpF	2.3	1.6	NS	NS	NS	NS
RprA	Activation of RpoS-dependent acid tolerance	NS	NS	1.3	2.5	1.7	2.8
RybA	Regulation of TyrR	2.4	1.3	NS	NS	NS	NS
RyeA	Possible regulator of OmpD	3.0	1.7	NS	NS	NS	NS
RyfD	Overexpression inhibits biofilm formation	2.4	1.3	NS	NS	NS	NS
RygD	Unknown	2.6	1.4	NS	NS	NS	NS
RygE	Unknown	2.6	1.4	NS	NS	NS	NS
RyhB	Iron homeostasis	3.5	1.5	3.3	1.6	NS	NS
RyjA	Upregulated in intracellular *Salmonella*	3.1	2.0	2.2	2.1	NS	NS
SroC	Overexpression inhibits biofilm formation	0.4	0.5	NS	NS	NS	NS
SroG	Phantom gene?	2.7	1.4	NS	NS	NS	NS
Tff	Probable attenuator of *rpsB* expression	2.2	1.4	NS	NS	NS	NS

a Differences in the abundances of the indicated sRNA species relative to the preinduction samples (CS1 and TS1). Only those sRNA species that exhibited ≥2-fold change (FDR adjusted *P* value of ≤0.05) for one or more samples are shown.

b Differences in the abundances of the indicated sRNA species in the production strain (TS2 and TS3) relative to those in the control strain (CS2 and CS3). Only those sRNA species that exhibited ≥2-fold change (unadjusted *P* value of ≤0.05) for one or more comparisons are shown.

c NS, not significant.

Two sRNAs (GadY and RprA) were differentially upregulated in the production strain compared to the control (Fig. 4 and Table 3). Both GadY and RprA sRNAs are associated with acid tolerance in E. coli (20, 21). GadY increases the stability of the *gadX* mRNA, and GadX, along with GadE and RcsB, activates expression of several components of the E. coli acid response. The E. coli acid fitness island consists of a 12-gene locus that contains the *gadE-mdtEF* operon that was upregulated in response to citramalate production (see above). Most of the genes in this island were also upregulated in response to citramalate, although most did not meet the criteria to be regarded as significant (see Fig. S5 in the supplemental material). Increased osmolarity enhanced RprA-dependent translation of the *rpoS* mRNA, and RprA is known to be upregulated when batch cultures are exposed to acetic, succinic, or itaconic acid (21, 22), suggesting that the enhanced abundance of RprA observed in the production fermentations may be a response to citramalate accumulation.

10.1128/mSystems.00187-19.5 FIG S5 Schematic representation of the regulation of the E. coli acid fitness island in response to citramalate production. Genes are represented as arrows with internal identifiers; DNAs coding for small regulator RNAs (sRNAs) are shown as triangles; mRNAs (black filled arrows), sRNAs (orange arrows), and proteins (ellipses) are also shown. The numbers show the difference (fold change) in either gene expression or protein abundance (red, upregulated; blue, downregulated) for the production fermentations versus control fermentations, with * indicating a significant change (≥2-fold; adjusted *P* value of ≤0.05). Shown is regulation (→, activation; ⊥, repression; →|, dual regulation) by GadY (black lines), GadW (brown lines), and GadX (purple lines). Increased expression of the sRNA, GadY, enhances translation of the transcription regulators GadW and GadX, resulting in upregulation of *gadAX*, *gadBC*, *gadE-mdtEF*, *hdeAB-yhiD*, *hdeD*, and *slp-dctR* genes or operons. The only protein of the acid fitness island detected in the proteome was Slp, which exhibited an ∼4-fold increase in abundance in the production strain. GadW mostly acts as a dual regulator of acid fitness island genes, and the lower activity predicted by TFInfer is consistent with enhanced abundance of GadY and activation of acid resistance genes by GadX (purple arrows). Download FIG S5, TIF file, 0.9 MB. Copyright © 2019 Webb et al. 2019 Webb et al. This content is distributed under the terms of the Creative Commons Attribution 4.0 International license.

### The chassis proteome responds to induction of recombinant protein production but is unperturbed by citramalate.

**FIG 5:**
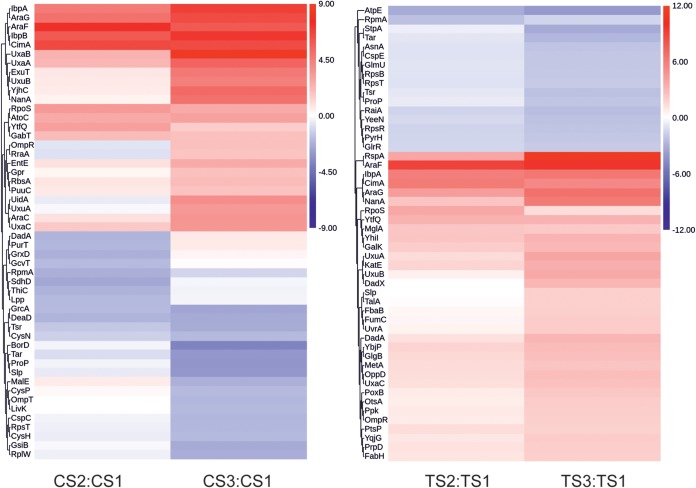
Heat map representations of changes in the proteomes of control (left) and production (right) strains. The columns show proteins that exhibited significant changes in abundance relative to the preinduction samples (CS1, TS1) and 4 (CS2, TS2) and 21.5 (CS3, TS3) h after induction of CimA3.7_dead_ (control) or CimA3.7 (production). Red represents increased abundance and blue decreased abundance. Protein identifiers are shown on the left.

To identify changes in the E. coli proteome in response to heterologous protein production, a label-free quantification approach was used to compare preinduction (CS1) and postinduction (CS2 and CS3) samples from the control fermentations expressing recombinant CimA3.7_dead_. Combining CS2 and CS3 revealed that 109 of the 1,413 detected proteins exhibited a change (64 increased, 45 decreased) in abundance postinduction (≥2-fold change in abundance relative to CS1; *P* < 0.05) (Fig. 5; Table S1D and Fig. S4). Of the 1,304 proteins that exhibited no significant change, 1,193 of the corresponding transcripts also did not significantly change. Fourteen differentially regulated genes and proteins were correlated (11 upregulated, *araG*, *betB*, *exuT*, *gabT*, *nanE*, *rspB*, *uxaB*, *uxaC*, *yafV*, *ycjX*, and *ygeA*; 3 downregulated, *cysD*, *purM*, and *pyrI*). The enhanced abundance of the arabinose transporter (AraG, 91.4-fold; AraF, 65.4-fold; and AraC, 12.4-fold) was expected in response to arabinose-mediated induction of *cimA3.7_dead_* expression, resulting in an ∼95-fold increase in CimA3.7_dead_ protein. A major feature of the transcript profiling was the downregulation of flagellar and chemotaxis genes, but this was not significant in the proteome. In both control and test fermentations, flagellar gene expression was downregulated after induction, and this is interpreted as a “fermentation” response to the production of recombinant protein and was not specific to citramalate production. Examination of the proteomic data for control and test fermentations showed that the only motility-related protein that was significantly downregulated after induction was Tar (8.3-fold and 6.2-fold, respectively). Nevertheless, all the other flagellum-related proteins that were detected exhibited lower abundance after induction (shown as protein, control fold decrease, test fold decrease: FliC, 2.7, 2.1; FliY, 2.9, 2.0; FlgK, 2.8, 2.7; FlgH, 2.0, 1.8; Tsr, 5.2, 4.9; CheA, 1.5, none; CheW, 1.2, none) but did not meet the criteria for statistical significance. In addition, not all flagellar proteins were detected in the proteomic analysis, and so changes in abundances of these could not be established. Thus, qualitatively the transcriptomics and proteomics of motility-related proteins are in agreement.

The *rspB* transcript and the RspA and RspB proteins were upregulated in both control and production strains upon induction of CimA. Co-overexpression of *rspAB* improved recombinant production of a model protein, β-galactosidase, by lowering accumulation of lactone by-products of amino acid metabolism and dysregulation of homoserine lactone-associated stationary-phase adaptations (23, 24). Thus, similar to the Ibp response (see below), upregulation of *rspAB* is likely associated with recombinant protein synthesis and not citramalate production.

Analysis in AmiGO of the 109 significantly regulated proteins at CS2 and CS3 indicated significant enrichment in uronic acid metabolism (GO:0006063; 23.23-fold; *P* = 0.006), monocarboxylic acid catabolic processes (GO:0072329; 5.29-fold; *P* = 0.0137) and organonitrogen compound biosynthesis (GO:1901566; 2.62; *P* = 0.0158).

The test fermentations, which produce both heterologous protein (active CimA3.7) and a chemical product (citramalate), also showed increased abundance of CimA3.7 (up to 76-fold), AraF (arabinose uptake), and UxaC and UxuAB (hexauronate catabolism) among a total of 152 proteins (74 more abundant and 80 less abundant from a total of 1,103 proteins detected; >2-fold; *P* < 0.05) (Fig. 5; Table S1E). Of the 951 proteins that exhibited no significant change, 944 also showed no changes at the level of transcription. The regulation of only four genes/proteins was positively correlated (*araG*, *ibpB*, *nanE*, and *rspB*).

In the context of recombinant protein production, the ∼100-fold increased abundances of the heat shock proteins IbpA and IbpB in both the control and test fermentations is of interest (Table S1D and E) (25). IbpA and IbpB have been also previously found as components of inclusion bodies formed during recombinant protein production and can act to stabilize loosely aggregated proteins and facilitate effective disaggregation by the DnaK and ClpB chaperone systems.

Differences (>2-fold, adjusted *P* value of <0.05) in protein abundance between samples TS2 and CS2 and TS3 and CS3 after normalization to the respective preinduction sample CS1 were observed for only five proteins: Slp (32-fold upregulated), YbjX (7.5-fold downregulated), OppD (5.4-fold upregulated), DeoA (5-fold upregulated), and Upp (4.8-fold upregulated) (Table S1F). Interestingly, the *slp* transcript, which encodes an outer-membrane-stabilizing protein that accumulates as growth rate decreases (26), was ∼3-fold upregulated when TS2 was compared to CS2, with an unadjusted *P* value of 0.016. No clear phenotypes have been associated with a *slp* mutant, which was unaltered in its resistance to hydrogen peroxide, ethanol, and SDS (26). There is evidence that Slp is enhanced in response to N limitation and temperature downshift and that it might be associated with resistance to low pH (27, 28). The relative upregulation of Slp at both the transcript and protein levels might suggest that this is a response specifically associated with citramalate production.

Overall, our data suggest that the chassis proteome is largely “blind” to citramalate accumulation as most reproducible changes appear related to protein production stress and occur under both test and control conditions without citramalate accumulation.

### Citramalate production did not alter the chassis lipidome, but strong growth cycle effects were observed.

**FIG 6:**
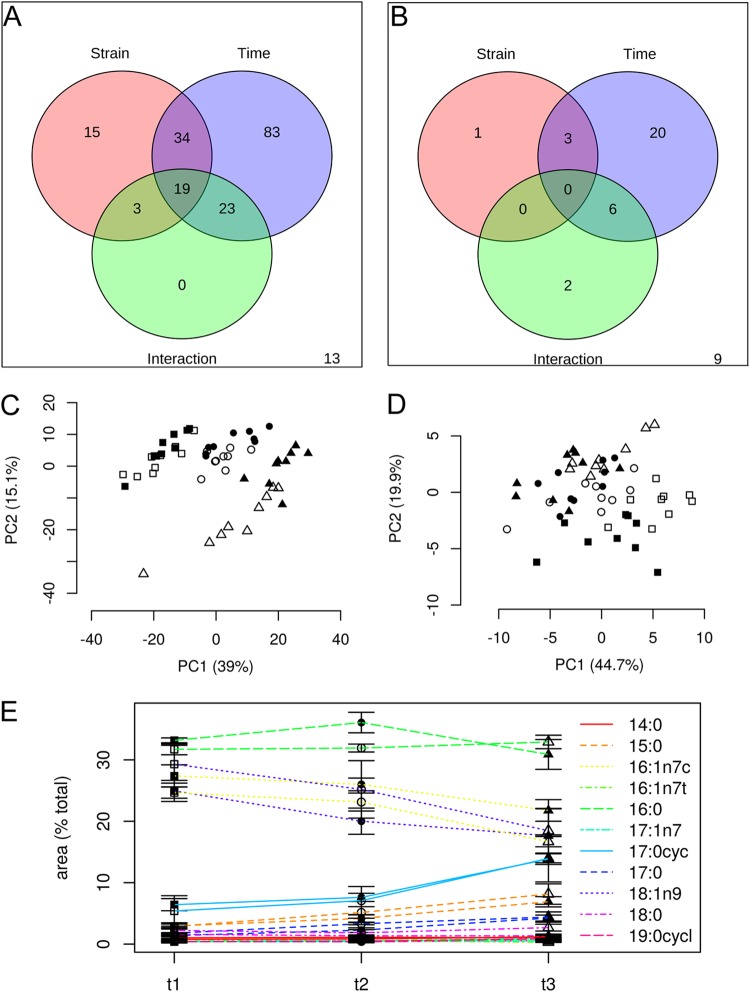
Lipid composition of the E. coli chassis changes as fermentation proceeds but is unaffected by citramalate production. Time series ANOVA2 on (A) positive LC-MS-MS intact lipid analysis indicated that the majority of lipid features changed with time rather than with citramalate production (labeled strain), with a more balanced output from negative LC-MS-MS data as shown in panel B. PCA indicated a clearer separation between the control strain (filled symbols) and production strain (open symbols) in positive mode (C) compared to negative mode (D). For both strains, there was a clear transition between preinduction (S1, squares) and postinduction (S2, circles; S3, triangles) samples. (E) Fatty acid methyl ester (FAME) analysis revealed equivalence between production and control strains, but in both a reciprocal decrease in 18:1n9 (dotted purple lines [filled symbols, control strain; open symbols, production strain]) and increase in the cyclopropanated 17:0cyc (solid blue lines [filled symbols, control strain; open symbols, production strain]) was observed over time.

The lipids present in the chassis (E. coli) membranes during high-cell density batch fermentation and citramalate production were determined by liquid chromatography (LC)-MS-MS with positive- and negative-mode heated electrospray ionization (HESI). In addition, gas chromatography (GC)-MS of fatty acid methyl esters (FAMES) was used to identify fatty acids present and any changes in the proportions of *cis*/*trans* isomers. LC-MS-MS analysis resulted in 190 peaks by positive ESI (see Table S3A in the supplemental material) and 41 by negative HESI (Table S3B). Chassis lipid composition was largely unaffected by production of citramalate but did change as both fermentations proceeded (Fig. 6A and B). Principal-component analysis (PCA) showed a separation between the control and test fermentations for the positive-mode ESI data, but this change was not evident in the negative-mode data set. The separation observed in the larger positive-mode data set arose from small changes in numerous lipids (Fig. 6C and D). Nevertheless, a minor increase in cyclopropane fatty acid content was observed for both fermentations in CS3 and TS3 (Fig. 6E). Although distinct changes in the E. coli lipidome were observed during the fermentation, these changes were not responses to citramalate production.

10.1128/mSystems.00187-19.8 TABLE S3 (A) Positive LC-MS-MS data for lipidome analysis. (B) Negative LC-MS-MS data for lipidome analysis. Download Table S3, XLSX file, 0.1 MB. Copyright © 2019 Webb et al. 2019 Webb et al. This content is distributed under the terms of the Creative Commons Attribution 4.0 International license.

### Citramalate excretion is likely to be nonspecific and mediated by several efflux systems.

The test fermentations yielded citramalate in the culture medium at a concentration of 25 g liter^−1^. As a charged molecule, citramalate secretion was anticipated to require the action of one or more efflux pumps. The efflux pump genes *alaE* and *mdtE* were upregulated after induction of CimA3.7, the *nmpC* gene coding for an outer membrane protein was downregulated, Slp an outer membrane-stabilizing protein, was significantly upregulated in the test fermentation proteome compared to the controls, and *acrA* appeared in both test and control fermentations as the gene most heavily influenced by MprA in the TFInfer analysis. Therefore, the corresponding mutant E. coli strains and the parent were transformed with pBAD24-*cimA3.7*, and small-scale citramalate production assays were performed using harvested cells. Analysis of the culture supernatants showed that the extracellular citramalate concentrations were similar for all strains tested (see Table S4 in the supplemental material). This suggested that individually *acrA*, *alaE*, *mdtE*, *nmpC*, and *slp* were not essential for citramalate excretion.

10.1128/mSystems.00187-19.9 TABLE S4 Deletion of genes possibly involved in citramalate efflux did not affect citramalate production in small-scale biotransformations. Biotransformations using the indicated strains were carried out as described. Glucose and citramalate concentrations in the media were measured by HPLC at the beginning and after 24 h of incubation. Errors are 95% confidence intervals (*n* = 3). Download Table S4, DOCX file, 0.01 MB. Copyright © 2019 Webb et al. 2019 Webb et al. This content is distributed under the terms of the Creative Commons Attribution 4.0 International license.

It is possible that intracellular concentrations of citramalate result in efflux as a result of low-affinity interactions with multiple transporters. Data for pyruvate excretion by E. coli were used to assess the likelihood of this suggestion (29). If the steady-state intracellular-to-extracellular pyruvate ratio of ∼50:1 (0.16 mM outside, 7.5 mM inside; dilution rate of 0.1 h^−1^; biomass, 2.5 g liter^−1^) results from a passive and unsaturated process (i.e., E. coli has no specific pyruvate efflux system), the rate of pyruvate export (6.4 × 10^−6 ^mol h^−1^ g^−1^) is equal to *k*[7.5 × 10^−3^], yielding an export rate constant *k* equal to 8.5 × 10^−4^ h^−1^. The maximum rate of citramalate appearance during the fermentations was ∼0.3 × 10^−3 ^mol h^−1^ g^−1^. Using the value for *k* calculated for pyruvate, the maximum intracellular concentration of citramalate is estimated to be ∼300 mM. The total E. coli metabolome concentration has been estimated at 300 mM (30). Assuming that E. coli is essentially “blind” to citramalate, as implied by the omic responses, then the percentage of total ion counts for citramalate (∼40%) suggests that the intracellular concentration reaches ∼200 mM, similar to the estimate calculated above and the measured extracellular citramalate concentration. These analyses suggested that citramalate concentrations inside and outside the cell are similar, which might be consistent with the operation of mechanosensitive channels opening in response to increased turgor pressure as the citramalate accumulates in the cytoplasm. However, citramalate production assays using harvested cells with single and double *mscL* and *mscS* mutations did not lower the extracellular concentration of citramalate, suggesting that citramalate export probably occurs through promiscuous major facilitator superfamily proteins when the intracellular citramalate concentration reaches ∼200 mM (Table S4).

### Conclusion.

**FIG 7:**
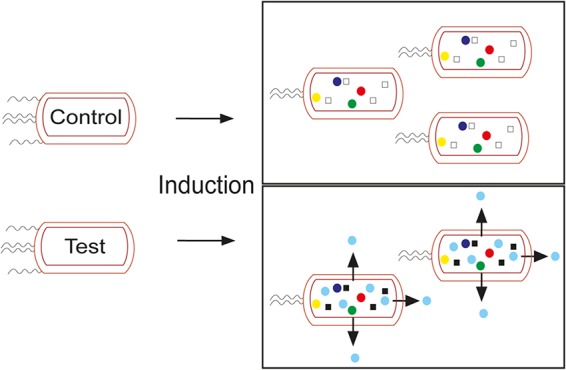
Schematic highlighting the key findings of this work. Overexpression of CimA3.7 (test, filled squares) or CimA3.7_dead_ (control, open squares) resulted in a downregulation of flagellar synthesis. The test fermentation overexpressing CimA3.7 produces citramalate (blue circles) in addition to the normal metabolome (multicolored circles), but at the expense of an equivalent amount of biomass production. Citramalate is exported through an undetermined route (arrows).

Characterization of the stresses experienced by bacterial cell factories during synthesis of recombinant proteins and chemical products is essential for improving process efficiency and designing new bacterial chassis. Many studies have investigated the potential use of engineered E. coli strains to produce native organic acids (e.g., lactate, malate, and succinate [31]). While high-titer processes for production of these organic acids have been developed, multiomic analyses of high-cell-density fed-batch cultures to determine the cellular responses during organic acid production are relatively few. Such information could be valuable in guiding further process and strain developments. Here, a comprehensive omic analysis of E. coli fed-batch fermentations for recombinant protein synthesis (CimA3.7_dead_) and for the production of a nonnative organic acid, citramalate, revealed minimal effects on gene expression, metabolism, or lipid composition, thus providing a simple explanation for the exceptional product titers and yields. Although it was anticipated that production of citramalate would require major rewiring of cellular metabolism rooted in reprogramming of transcription and consequent changes in the proteome and lipidome, multiomic analyses revealed that few adaptations were required to permit high production of citramalate (Fig. 7).

The multiomic analysis of E. coli high-cell-density batch cultures expressing inactive recombinant CimA3.7_dead_ (∼20 mg g^−1^ cdw) permitted identification of core stress responses associated with recombinant protein expression (Fig. S1B). Transcriptomic and proteomic analyses indicated that recombinant protein expression was associated with downregulation of flagellar genes and induction of the *ibpAB*-encoded chaperones. It has been shown that induced expression of the *ibpAB* operon can be a useful intervention to enhance recombinant protein yields, especially when combined with other chaperones, such as ClpB (25, 32, 33).

As expected, the postinduction exo- and endo-metabolite profiles of the production strain were dominated by citramalate. Two minor by-products, citraconate and 2-ethylmalate, were detected at concentrations that would not warrant further intervention to eliminate their presence. During citramalate production, the transcript, protein, and lipid profiling indicated that the level of stress imposed on the E. coli chassis was low, but some responses were detected that could be attributed to the citramalate synthesis, including roles for sRNA molecules in maintaining carbon flux through glycolysis (CsrB) and in acid tolerance (GadY and RprA). Optimizing these sRNA responses could represent a possible intervention to further enhance citramalate yields. The analyses of citramalate production reported here suggest that E. coli is a robust chassis organism and is likely to be an excellent choice for production of other organic acids.

## MATERIALS AND METHODS

### Growth media.

Escherichia coli strains were grown in either LB (Melford, United Kingdom), SM or ML media at 37°C. ML medium (1 liter) contained MgSO_4_·7H_2_O (2 ml; 246.47 g liter^−1^), salt solution (200 ml) containing K_2_HPO_4_ (73 g liter^−1^), NaH_2_PO_4_·2H_2_O (18 g liter^−1^), (NH_4_)_2_SO_4_ (10 g liter^−1^), ammonium citrate (2.5 g liter^−1^), trace elements (2 ml) containing Na_2_EDTA·2H_2_O (22.3 g liter^−1^), FeCl_3_ (10.03 g liter^−1^), CaCl_2_·2H_2_O (0.5 g liter^−1^), ZnSO_4_·7H_2_O (0.18 g liter^−1^), CoCl_2_·6H_2_O (0.18 g liter^−1^), CuSO_4_·5H_2_O (0.16 g liter^−1^), and MnSO_4_·H_2_O (0.15 g liter^−1^) and glucose (the concentration of which, as indicated in the text, was added from a sterile stock solution at 250 g liter^−1^). When required, carbenicillin was added to the medium at a concentration of 50 mg liter^−1^. Polypropylene glycol (0.1 ml liter^−1^) was added to the medium when used in a fermentor. The SM medium (1 liter [34]) contained glycerol (30 ml; 500 g liter^−1^), yeast extract (50 ml; 100 g liter^−1^), MgSO_4_ (2 ml; 246.47 g liter^−1^), salt solution (200 ml) containing Na_2_HPO_4_·12H_2_O (75.6 g liter^−1^), KH_2_PO_4_ (15 g liter^−1^), NH_4_Cl (5 g liter^−1^), NaCl (2.5 g liter^1^), CaCl_2_ (55 mg liter^−1^), and SM trace element solution (100 μl) containing FeSO_4_·7H_2_O (80 g liter^−1^), AlCl_3_·6H_2_O (10 g liter^−1^), MnSO_4_·H_2_O (10 g liter^−1^), CoCl_2_ (4 g liter^−1^), ZnSO_4_·7H_2_O (2 g liter^−1^), Na_2_MoO_4_·2H_2_O (2 g liter^−1^), CuCl_2_·2H_2_O (1 g liter^−1^), and H_3_BO_3_ (0.5 g liter^−1^) dissolved in HCl (3 M). Glycerol, yeast extract, MgSO_4_·7H_2_O and salt solutions were prepared and autoclaved separately. Trace elements were sterilized by filtration using 0.2-μm-pore filters. All solutions were cooled before mixing.

### Preparation of E. coli strains.

Escherichia coli BW25113 and E. coli BW25113 *ldhA* were purchased from the Keio collection (35). A control plasmid (pBAD-*cimA3.7_dead_*) expressing an inactivated CimA3.7 by virtue of a His192Ala substitution was constructed using Quickchange II XL site-directed mutagenesis kit (Agilent Technologies). The manufacturer’s protocol was followed with primers (5′-ACCTGCCGGTTAGCGTGGCCTGCCATAACGATTTCGGC-3′ and 5′-GCCGAAATCGTTATGGCAGGCCACGCTAACCGGCAGGT-3′) and pBAD-*cimA3.7* (5) as the template. Electro-competent E. coli BW25113 *ldhA* was transformed to create strains expressing active or inactive CimA; transformants were selected on LB agar supplemented with carbenicillin (50 mg liter^−1^). Escherichia coli BW25113 *ldhA* was used in the fermentation processes to minimize lactate formation based on unpublished results (J. Webb, personal communication). Other E. coli BW25113 mutants were also obtained from the Keio collection, except the *mscL mscS* double mutant, which was constructed using pCP20 (36) to cure the kanamycin cassette from E. coli BW25113 *mscL*::Kan^r^, followed by deletion of *mscS* from the resulting strain (E. coli BW25113 *mscL*::FRT) by P1 transduction of the *mscL* mutation from E. coli BW25113 *mscL*::Kan^r^.

### Citramalate production using harvested cells.

Single colonies of E. coli BW25113 *ldhA* expressing either active or inactive CimA3.7 were grown overnight (37°C, 250 rpm) in SM glycerol medium. Cultures were diluted to a starting OD_600_ of 0.1 in SM glycerol medium, and the culture was incubated (250 rpm, 37°C) until the OD_600_ reached 0.6. CimA3.7 expression was induced by the addition of l-arabinose (0.2 g liter^−1^). Cells were harvested by centrifugation (4,000 × *g*, 20 min, 4°C) 4 h postinduction (OD_600_ of 3 to 4) and concentrated to a dry cell weight of 15 g liter^−1^ in SM medium without NH_4_Cl or yeast extract but with glucose (20 g liter^−1^). The cell suspensions (approximately 10 ml) were incubated in baffled flasks (250 ml, 24 h, 250 rpm, 37°C) before analysis of culture supernatants by high-pressure liquid chromatography (HPLC).

### Fermentations.

Inocula for fed-batch fermentations were produced by inoculating E. coli BW25113 *ldhA* pBAD24-*cimA3.7* or E. coli BW25113 *ldhA* pBAD24-*cimA3.7_dead_* into ML medium (50 ml) supplemented with glucose (10 g liter^−1^) and carbenicillin (50 μg ml^−1^) and incubating overnight (200 rpm, 37°C). The cultures were diluted to OD_600_ of 0.1 in sterile water (50 ml) and used to inoculate ML medium (1 liter) supplemented with glucose (11.9 g liter^−1^) and carbenicillin in a 3-liter BioFlo/CelliGen 115 bioreactor (New Brunswick Scientific). Cultures were grown at 37°C, and the pH was maintained at 7.0 ± 0.1 by the addition of NH_4_OH (28 to 30%) and 2 M H_2_SO_4_ (2 M). The airflow rate was initially set at 1 liter min^−1^, and the dissolved oxygen (dO_2_) was maintained at 30% of saturation by automatic control of stirrer speed between 400 and 1,200 rpm and an airflow cascade between 1 and 7 liter min^−1^. The airflow cascade was only implemented when maximum agitation was reached. Drops of polypropylene glycol (100%) were manually added to the medium when required to avoid foaming. When the glucose in the batch medium had been consumed, indicated by a sharp increase in dO_2_ and confirmed using glucose test strips (117866; Merck Millipore), a feed of glucose was started containing glucose (650 g liter^−1^), yeast extract (5.9 g liter^−1^), MgSO_4_ (7.2 g liter^−1^), and trace elements (11.8 ml). The flow rate was adjusted manually as required to maintain a pseudoexponential growth rate of approximately 0.25 h^−1^ and avoid the accumulation of excess glucose in the culture, using the test strips as confirmation as described above. Protein expression was induced by the addition of l-arabinose (0.02% wt/vol) when the culture OD_600_ was 50.

### Analytical methods.

Growth was monitored by measuring OD_600_. Samples were diluted in deionized water when the OD_600_ was >0.8. Dry cell weight was measured by centrifuging 1-ml samples in preweighed polypropylene tubes, removing the supernatant, and drying the pellets to a constant weight. d-Glucose, (*R*)-citramalate, and other organic acids were quantified by HPLC using an Agilent 1200 series HPLC system equipped with both UV (215 nm) and refractive index detectors. Samples were resolved using a Rezex ROA organic acid H+ column (Phenomenex) at 55°C with 0.01 N H_2_SO_4_ (0.5 ml min^−1^) as the mobile phase. Samples were prepared for HPLC analysis by centrifuging (12,000 × *g*, 5 min) and filtering the supernatants (0.2-μm-pore filter). Data analysis was performed with ChemStation software using calibration curves prepared using authentic standards of each compound (0.1 to 200 mM).

### Transcriptomics.

Fermentation samples (3 × 0.1 ml) were mixed with RNAprotect (Qiagen [0.2 ml]), vortexed, and incubated at room temperature (5 min). The mixture was then centrifuged (16,000 × *g*, 2.5 min, 4°C) and the supernatant discarded, and pellets were stored at −80°C. Cell pellets were thawed at room temperature and normalized in TE buffer (10 mM Tris-HCl at pH 8.0, 0.1 mM EDTA), such that 1 ml of TE buffer contained 2 OD_600_ units. Aliquots (1 ml) were harvested by centrifugation (12,000 × *g*, 1 min), and the pellets were resuspended in 100 μl TE buffer containing lysozyme (15 mg ml^−1^). These were incubated at room temperature (10 min) with vortexing every 2 min. Total RNA was prepared using the RNeasy RNA purification kit (Qiagen) according to the manufacturer’s protocol (including the on-column DNase treatment step). Labeled cDNA was produced using SuperScriptIII reverse transcriptase (Invitrogen) with the Cy3-dCTP included in the dNTP mixture. Labeled E. coli genomic DNA was produced using the BioPrime DNA labeling kit (Invitrogen) with Cy5-dCTP included in the dNTP mixture. Labeled genomic DNA and cDNA were combined and hybridized overnight to an oligonucleotide microarray (Agilent Technologies). Quantification of cDNA, hybridization of cDNA to microarrays, microarray processing, and microarray scanning were performed as described in the Fairplay III labeling kit (Agilent Technologies, 252009, version 1.1). Microarrays were scanned with a high-resolution microarray scanner (Agilent Technologies). Features with background intensities exceeding 10 times the array median, or with a signal/background ratio below 3 were excluded from further analysis. Background correction (37), within-array Loess normalization (38), and between-array quantile normalization were applied to the remaining features using the R statistical package LIMMA from Bioconductor (39). Moderated *t* statistics were calculated using gene-wise linear models with an empirical Bayes approach (40, 41). *P* values were adjusted for multiple testing using the Benjamini-Hochberg method (42). Transcripts exhibiting ≥2-fold change in abundance with an adjusted *P* value of <0.05 were deemed to be differentially regulated. The data are available in ArrayExpress. The relative activities of transcription factors were inferred using the TFInfer software package as previously described (14).

### Metabolomics.

Quenching of fermentation samples (0.5 ml) was performed by mixing ethanol solution (40% vol/vol) prepared in NaCl (0.8% wt/vol) at −20°C (43). The mixture was centrifuged (16,000 × *g*, 2.5 min, 4°C). The supernatant was discarded, and the pellets were stored (−80°C) until required. Cell pellets were resuspended to an OD_600_ of 1 in chloroform-methanol (1:1, −20°C), and 1 ml was transferred to a fresh polypropylene tube. An ice-cold ball bearing was added to each tube before incubation (−80°C, 1 h). The samples were then vortexed twice (30 s) and returned to the −80°C freezer for 1 h. Milli-Q water (400 μl) was added to each sample, followed by vortex mixing (30 s). Samples were then centrifuged (4,000 × *g*, 1 min, 4°C). The upper layer was transferred to a fresh tube. This step was repeated, and the two organic phases were combined. Electrospray ionization time-of-flight (ESI-TOF) MS was performed on a Hybrid quadrupole time-of-flight (TOF) LC-MS-MS spectrometer (Waters, Ltd., Manchester, United Kingdom) based on the methods described by Davey et al. (44) and Walker (45). Data acquisition and processing were performed on MassLynx (version 4) to create centroid peak lists (*m*/*z* accurate to 4 decimal places versus ion counts), which were then transferred to Microsoft Excel (Microsoft Corp., USA) as text files. The mass spectrometer was operated in negative-ion mode at a rate of 1 scan per s for 6 min. Samples were loaded using a syringe pump (Razel, CT, USA) at a flow rate of 20 μl min^−1^. A Lockspray interface was used to give an external standard and allow automated correction of mass measurements (5 ng liter^−1^ sulfadimethoxine, giving a lock mass of 309.0653). Samples were analyzed in a randomized order to minimize effects of day-to-day machine variation. Data processing and downstream analysis were performed in R, using Bioconductor package XCMS (46). Peaks were aligned across analytical replicates and grouped into 0.2-*m/z*-width bins (47). Peaks were rejected if all three replicates were not present or the mass variance fell outside an acceptable range defined as a function of the *m/z* (formula modified from Overy et al. [48]; H. Walker, personal communication).

### Proteomics. (i) Protein extraction and peptide sample preparation.

Fermentation samples (500 μl) were harvested by centrifugation (16,000 × *g*, 2.5 min, 4°C). The supernatant was discarded and the pellet stored (−80°C). Cells were embedded in a 20% polyacrylamide gel matrix and digested and washed following the gel-aided sample preparation (GASP) protocol with three minor changes (49). First, samples were reduced using 10 mM Tris(2-carboxyethyl)phosphine (TCEP); second, they were desalted after gel extraction on Sola C_18_ cartridges following the manufacturer’s instructions; third, after the samples were dried to near dryness, they were resuspended in 0.1% formic acid for injection into the mass spectrometry-liquid chromatography system. Injections were normalized by a quantitative colorimetric assay (Thermo) that uses a modified bicinchoninic acid (BCA) chemistry for sensitive and accurate peptide sample quantification to keep samples comparable and to optimize signal intensity.

The buffers used for the GASP method were as follows. Lysis was performed on ice (30 min) in a buffer containing urea (6 M), thiourea (1.5 M), SDS (4%), TCEP (10 mM), 20 mM Tris (20 mM, pH 8.0). Alkylation was achieved on ice (30 min) using an equal volume of monomeric acrylamide mix solution (40%, 37.5:1 acrylamide-bisacrylamide solution). Polymerization was initiated at room temperature by addition of *N*,*N*,*N*′,*N*′-tetramethylethylenediamine (TEMED [5 μl]) and ammonium persulfate (APS [10%]) followed by rapid mixing by vortexing. After polymerization was complete (∼30 min), gel plugs were cut by centrifugation through a membrane-less SpinX centrifugation filter support device (Costar) to achieve identical sizes of gel pieces. Gel pieces were fixed by addition of fixation buffer (1 ml) containing methanol-acetic acid-water (50:40:10) and overhead rotation (10 min). After brief pulse centrifugation, the supernatant was discarded and gel pieces were rehydrated with urea (6 M, 0.5 ml, 10 min). Acetonitrile (1 ml) was used to dehydrate gel pieces. Following removal of the supernatant using a gel loading pipette tip (200-μl-volume pipette tip with a long narrow end to avoid loss of gel pieces), two more rounds of urea and acetonitrile washes were performed to minimize carryover of SDS and other contaminants. After these washes, the gel pieces were pH adjusted using triethylammonium bicarbonate (TEAB [500 μl, 50 mM, pH 8.0]) and rotation (10 min). Gel pieces were dehydrated once more using acetonitrile (1 ml), supernatant was removed, and acetonitrile (0.5 ml) was used for more thorough dehydration of the gel pieces. Dried-out gel pieces were soaked with trypsin solution (1/50 enzyme/substrate ratio based on expected protein content from known cell mass in Tris buffer [20 mM, pH 8.0]). Digestion was performed overnight with shaking (1,000 rpm, 37°C). Elutions were performed using one gel volume: elution 1 with acetonitrile, 2 with formic acid (5%), and 3 with acetonitrile. Elutions 2 and 3 were combined.

### (ii) Mass spectrometry analysis.

Peptides (1 μg) from each label-free sample were injected and separated via 1D-ultrahigh-pressure liquid chromatography (UHPLC) using a Dionex Ultimate 3000 RSLC nanoUPLC (Thermo Fisher Scientific, Inc., Waltham, MA, USA) system, and masses were analyzed using an Orbitrap Fusion Lumos mass spectrometer (Thermo Fisher Scientific, Inc., Waltham, MA, USA). Separation of peptides was performed by reverse-phase chromatography at a flow rate of 300 nl min^−1^ on a Thermo Scientific reverse-phase nano-Easy-spray column (Thermo Scientific PepMap C_18_, 2-μm particle size, 100-Å pore size, 75-μm inside diameter [i.d.] by 50-cm length). Peptides were loaded onto a precolumn (Thermo Scientific PepMap 100 C_18_, 5-μm particle size, 100-Å pore size, 300-μm i.d. by 5-mm length) from the Ultimate 3000 autosampler with formic acid (0.1%) for 3 min at a flow rate of 10 μl min^−1^. After this period, the column valve was switched to allow elution of peptides from the precolumn onto the analytical column. Solvent A was H_2_O containing formic acid (0.1%), and solvent B was acetonitrile (80%), H_2_O (20%), and formic acid (0.1%). The linear gradient employed was 2 to 45% B in 90 min. The total run time was 120 min, including a high-organic-wash step and reequilibration step. Peptides were transferred to the gaseous phase with positive-ion electrospray ionization at 2.1 kV. In DDA, the top 20 precursors were acquired between 360 and 1,500 *m*/*z* with a 1.5-Th (Thomson) selection window, dynamic exclusion of 35 s, normalized collision energy (NCE) of 30%, and resolutions of 70,000 for MS and 17,500 for MS2; the automatic gain control (AGC) target was 500,000. The .raw files were analyzed with MaxQuant version 1.5.5.1 using default settings. The minimal peptide length was set to 6. Trypsin/P was used as the digestion enzyme. Search criteria included propionamidation of cysteine (PAM-Cys [+71.0371 Da]) as a fixed modification and oxidation of methionine and acetyl (protein N terminus) as variable modifications. Up to two missed cleavages were allowed. The mass tolerances for the precursor were 20 and 4.5 ppm for the first and the main searches, respectively, and that for the fragment ions was 20 ppm. The files were searched against the E. coli K-12 reference UniProt fasta database (July 2017 [4,306 sequences]). The identifications were filtered to obtain a false-discovery rate (FDR) of 1% at the peptide and the protein levels. No filter was applied to the number of peptides per protein. Log_2_-transformed quantification results were tested for differential abundance using moderated *t* statistics using the LIMMA R package. The mass spectrometry proteomics data have been deposited into the ProteomeXchange Consortium via the PRIDE partner repository (50).

### Lipidomics.

Fermentation samples (500 μl) were harvested by centrifugation (16,000 × *g*, 2.5 min, 4°C), and the supernatants were discarded. The pellet was washed twice in a salt solution consisting of (NH_4_)SO_4_ (2 g liter^−1^), K_2_HPO_4_ (14.6 g liter^−1^), NaH_2_PO_4_·2H_2_O (3.6 g liter^−1^), and (NH_4_)_2_citrate (0.5 g liter^−1^). Samples were lyophilized, and 4 mg was transferred into 2-ml microcentrifuge tubes for lipid extraction. All solvents were HPLC grade from Rathburn Chemicals (Walkerburn, Scotland, United Kingdom). Lipids were extracted by adding isopropanol (400 μl) and a deuterated internal standard mix (10 μl SPLASH lipidomix, p/n 330707; Avanti Polar Lipids, AL, USA). Samples were briefly resuspended with a micropestle, and the tubes were capped and incubated (95°C, 15 min) to inactivate phospholipases. Samples were cooled (4°C) before adding hexane (600 μl), followed by brief vortex mixing and centrifuging (16,000 × *g*, 10 min). The supernatant was transferred to a fresh tube and aqueous Na_2_SO_4_ (500 μl, 6.7% wt/vol) added to promote phase separation. The tubes were briefly vortexed and centrifuged to separate the layers, and the organic (top) layer (200 μl) removed to a glass HPLC vial for LC-MS analysis of intact lipids. A separate aliquot (200 μl) was kept for transmethylation to fatty acid methyl esters (FAMEs) for analysis by GC-MS. All samples were dried on a centrifugal evaporator. Technical triplicates were extracted from each fermentor at each time point, and extraction blanks were processed in parallel to correct for any background contamination in subsequent analyses.

For GC-MS analysis, FAMEs were generated by incubating dried samples with 1 N methanolic HCl (Supelco) in sealed glass vials (85°C, 4 h). Samples were cooled before adding hexane (200 μl) and aqueous KCl (250 μl 0.9%), vortexing and centrifuging briefly, and collecting the upper phase (100 μl) for analysis. GC-MS analysis of FAMEs was performed using an Agilent 6890 gas chromatograph interfaced with a Leco Pegasus IV TOF MS (Leco, Stockport, United Kingdom) system. Data acquisition and processing was performed using ChromaTof 4.5 software (Leco). A 1-μl aliquot was injected in pulsed splitless mode onto an Rxi-5Sil MS column (30-m by 0.25-mm i.d. by 0.25-μm film thickness; Thames Restek, Saunderton, United Kingdom). FAMEs were separated in He at a constant flow of 1 ml min^−1^ under the following conditions: initial 100°C for 2 min, linear ramp at 5°C min^−1^ to 300°C, hold at 300°C for 2 min, and total run time of 44 min. EI spectra were obtained at −70 eV at a detector voltage of 1,790 V, at a rate 5 spectra s^−1^ over the *m/z* range 50 to 450. ChromaTof was used to detect peaks at a minimum s/n of 10 and peak width of 3 s, and peak areas were reported from deconvoluted total-ion chromatograms. Peaks were identified by searching electron ionization (EI) spectra against the NIST 2014 library and comparing retention times and spectra to a bacterial FAME reference mix (Larodan, Sweden).

For LC-MS analysis, samples were reconstituted in acetonitrile-isopropanol (200 μl, 70:30 vol/vol). LC was achieved using a Waters Acquity I-Class UPLC system, fitted with an Accucore C_30_ column (Thermo Scientific [100 mm by 2.1 mm, 2.6-μm particle size]). The column was protected with an Accucore C_30_ guard cartridge (10 mm by 2.1 mm). Samples were chilled (10°C) in the UPLC autosampler, and a 2-μl aliquot was injected. The weak wash solvent was methanol (5% vol/vol), and the strong wash solvent was isopropanol. For sample elution, the mobile phase A was acetonitrile-water (60:40 vol/vol) plus ammonium formate (10 mM) plus formic acid (0.1% vol/vol). Mobile phase B was acetonitrile-isopropanol (10:90 vol/vol) plus ammonium formate (10 mM) plus formic acid (0.1% vol/vol). Lipids were eluted at 0.35 ml min^−1^ and 40°C, using the following gradient: initial 1% B, linear increase to 99% B at 21 min, hold at 99% B until 24 min, return to initial conditions at 24.1 min, and hold at initial conditions until 28 min. Mass spectra were acquired using a Thermo Orbitrap Fusion Tribrid mass spectrometer, fitted with a heated electrospray ionization (HESI) ion source. The UPLC and MS were controlled by Thermo Xcalibur 4.0 software. Alternate injections were made in positive- and negative-ion acquisition modes. In positive mode, the spray voltage was set to 3,500 V, and in negative mode, it was set to 3,000 V. In both modes, nitrogen gas flows for sheath, auxiliary, and sweep gases were set to 42, 14, and 1 arbitrary unit, respectively. The ion transfer tube was set to 300°C and the HESI vaporizer to 280°C. Data were acquired in a set 1-s cycle time, with high resolution (240,000 full width at half-maximum [FWHM] at *m*/*z* 200) MS1 profile spectra acquired over the *m*/*z* 200 to 1,600 scan range, with an AGC target of 200,000 and a maximum ion injection time of 100 ms. MS1 data were internally calibrated during acquisition to <1-ppm mass error using the installed Thermo Easy IC option. Data-dependent MS2 scans were collected in parallel using a 1-*m*/*z* quadrupole isolation window and scanned at approximate unit mass resolution in centroid mode from the ion trap, alternating between higher-energy collisional dissociation (HCD) and collision-induced dissociation (CID) fragmentation modes. Only MS1 masses of >400 *m*/*z* were selected for fragmentation, with an AGC target of 10,000 and max ion time of 50 ms. HCD spectra were collected in stepped collision energy mode centered at 40% and CID spectra at a fixed 40%. Dynamic exclusion was enabled, with exclusion after *n* = 1 times, for 6 s.

For analysis of LC-MS-MS data, Xcalibur .raw files were converted to centroided. mzML format using the msconvert function of ProteoWizard (release 3.0.18114). Peak processing workflows were conducted in R 3.5.0. MS1 peaks were extracted using the “centWaveWithPredictedIsotopeROIs” method from the XCMS package (version 3.2.0 [46, 51]). Peaks were grouped across samples, missing peaks imputed by reintegration within group boundaries, and group median *m*/*z* values further processed using the CAMERA package (version 1.23.3 [52]), to identify isotopes and adducts. Candidate formulas were generated with adapted code from the rcdk package (version 3.4.9 [53]), with the following limits: positive mode, C10-300, H20-500, O0-20, N0-3, P0-2, Na02, RDBE −0.5-18, 2-ppm error; negative mode, C10-300, H20-500, O0-20, N0-3, P0-2, S0-2, RDBE −0.5-18, 20-ppm error. Consensus peak groups were then filtered with custom R scripts to (i) exclude any peak groups where any peak in the group was present with an area less than the mean + 3 standard deviations of the value from blank extracts, (ii) only retain the most intense monoisotopic peak identified by CAMERA, and (iii) only retain peaks with valid molecular formulas. MS1 peaks were searched against downloaded local copies of the E. coli metabolome database (ECMDB [54]) and the Lipid Maps Structure Database (LMSD [http://www.lipidmaps.org]). Consensus HCD and CID MS2 spectra were extracted with custom scripts and searched against the *in silico* LipidBlast (55) and LipidMatch (56) databases. Peaks were annotated following manual examination of MS2 spectra in consensus with returned database hits. All retained peaks were normalized to the SPLASH deuterated PC [15:0_18:1(d7)] internal standard and then to sample dry weight. Statistical analyses were carried out on glog normalized data (lambda value in glog transform taken to be 1/10 of nonzero minimum), using time-series ANOVA2 models from the online MetaboAnalyst resource (https://www.metaboanalyst.ca).

### Citramalate synthase assay.

Cell-free extracts of E. coli BW25113 *ldhA* pBAD24-*cimA3.7*, E. coli BW25113 *ldhA* pBAD24-*cimA3.7A_dead_* or E. coli BW25113 *ldhA* were prepared from cultures (400 ml) 4 h after induction with l-arabinose (0.02 g liter^−1^). Cells were harvested by centrifugation (8,000 × *g*, 10 min, 4°C) and resuspended in HEPES buffer (0.1 M, pH 7.5) containing MgCl_2_ (5 mM). Cells were lysed by three passages through a French pressure cell. The resulting cell suspension was centrifuged (12,000 × *g*, 10 min), and the supernatant containing the soluble protein was filtered (0.2-μm-pore filter). Total protein concentration was estimated using a Bio-Rad protein assay as described by the manufacturer, and the protein content was normalized to 1 mg ml^−1^ in HEPES buffer (0.1 M, pH 7.5) containing MgCl_2_ (5 mM). CimA3.7 and CimA3.7_dead_ were assayed using a modified method described by Howell et al. (57). The enzyme assay mixture (1 ml) contained acetyl-CoA (1 mM), pyruvate (1 mM), cell extract (200 μl), and HEPES buffer (0.1 M, pH 7.5) containing MgCl_2_ (5 mM). The reaction mixture was incubated at 37°C, and at regular intervals (10 min), samples (100 μl) were taken and mixed with a sample analysis mixture (900 μl), and the absorbance at 412 nm was measured. The sample analysis mixture (900 μl) contained 5,5′-dithio-*bis*-(2-nitrobenzoic acid) (DTNB [0.56 mM]), Tris-HCl (78 mM, pH 8.0), and distilled water (dH_2_O). Where indicated in the text, pyruvate was replaced by 2-oxobutyrate.

### Data availability.

Transcriptomics data are available in ArrayExpress under accession no. E-MTAB-7257. The mass spectrometry proteomics data have been deposited in the ProteomeXchange Consortium database via the PRIDE partner repository under accession no. PXD013088 (50).

## References

[1] Carlsson M, Habenicht C, Kam LC, Antal MJJ, Bian N, Cunningham RJ, Jones MJ 1994 Study of the sequential conversion of citric to itaconic to methacrylic acid in near-critical and supercritical water. Ind Eng Chem Res 33:1989–1996. doi:10.1021/ie00032a014.

[2] Johnson DW, Eastham GR, Poliakoff M, Huddle TA 2012 Process for production of methacrylic acid, methacrylic acid derivatives and polymers. WIPO patent application WO2012/069813.

[3] Atsumi S, Liao JC 2008 Directed evolution of *Methanococcus jannaschii* citramalic acid synthase for biosynthesis of 1-propanol and 1-butanol by *Escherichia coli*. Appl Environ Microbiol 74:7802–7808. doi:10.1128/AEM.02046-08. 18952866 PMC2607174

[4] Eastham GR, Johnson DW, Archer I, Carr R, Webb J, Stephens G 2015 A process for production of methacrylic acid and derivatives thereof. WIPO patent application WO/2015/022496.

[5] Webb JP, Arnold SA, Baxter S, Hall SJ, Eastham G, Stephens G 2018 Efficient bio-production of citramalic acid using an engineered *Escherichia coli* strain. Microbiology 164:133–141. doi:10.1099/mic.0.000581. 29231156 PMC5882075

[6] Parimi NS, Durie IA, Wu X, Niyas MM, Eiteman MA 2017 Eliminating acetate formation improves citramalic acid production by metabolically engineered *Escherichia coli*. Microb Cell Fact 16:114. doi:10.1186/s12934-017-0729-2. 28637476 PMC5480221

[7] Wu X, Eiteman MA 2016 Production of citramalic acid by metabolically engineered *Escherichia coli*. Biotechnol Bioeng 113:2670–2675. doi:10.1002/bit.26035. 27316562

[8] Wu X, Eiteman MA 2017 Synthesis of citramalic acid from glycerol by metabolically engineered *Escherichia coli*. J Ind Microbiol Biotechnol 44:1483–1490. doi:10.1007/s10295-017-1971-7. 28744578

[9] Carbon S, Ireland A, Mungall CJ, Shu S, Marshall B, Lewis S, AmiGO Hub, Web Presence Working Group. 2009 AmiGO: online access to ontology and annotation data. Bioinformatics 25:288–289. doi:10.1093/bioinformatics/btn615. 19033274 PMC2639003

[10] Marisch K, Bayer K, Scharl T, Mairhofer J, Krempl PM, Hummel K, Razzazi-Fazeli E, Striedner G 2013 A comparative analysis of industrial *Escherichia coli* K-12 and B strains in high-glucose batch cultivations on process-, transcriptome- and proteome level. PLoS One 8:e70516. doi:10.1371/journal.pone.0070516. 23950949 PMC3738542

[11] Dürrschmid K, Reischer H, Schmidt-Heck W, Hrebicek T, Guthke R, Rizzi A, Bayer K 2008 Monitoring of transcriptome and proteome profiles to investigate the cellular response of *E. coli* towards recombinant protein expression under defined chemostat conditions. J Biotechnol 135:34–44. doi:10.1016/j.jbiotec.2008.02.013. 18405993

[12] Mairhofer J, Scharl T, Marisch K, Cserjan-Puschmann M, Striedner G 2013 Comparative transcription profiling and in-depth characterization of plasmid-based and plasmid-free *Escherichia coli* expression systems under production conditions. Appl Environ Microbiol 79:3802–3812. doi:10.1128/AEM.00365-13. 23584782 PMC3675926

[13] Singh AB, Sharma AK, Mukherjee KJ 2012 Analyzing the metabolic stress response of recombinant *Escherichia coli* cultures expressing human interferon-beta in high cell density fed batch cultures using time course transcriptomic data. Mol Biosyst 8:615–628. doi:10.1039/C1MB05414G. 22134216

[14] Asif HM, Rolfe MD, Green J, Lawrence ND, Rattray M, Sanguinetti G 2010 TFInfer: a tool for probabilistic inference of transcription factor activities. Bioinformatics 26:2635–2636. doi:10.1093/bioinformatics/btq469. 20739311

[15] Gama-Castro S, Salgado H, Santos-Zavaleta A, Ledezma-Tejeida D, Muñiz-Rascado L, García-Sotelo JS, Alquicira-Hernández K, Martínez-Flores I, Pannier L, Castro-Mondragón JA, Medina-Rivera A, Solano-Lira H, Bonavides-Martínez C, Pérez-Rueda E, Alquicira-Hernández S, Porrón-Sotelo L, López-Fuentes A, Hernández-Koutoucheva A, Del Moral-Chávez V, Rinaldi F, Collado-Vides J 2016 RegulonDB version 9.0: high-level integration of gene regulation, coexpression, motif clustering and beyond. Nucleic Acids Res 44:D133–D143. doi:10.1093/nar/gkv1156. 26527724 PMC4702833

[16] Xiao M, Zhu X, Xu H, Tang J, Liu R, Bi C, Fan F, Zhang X 2017 A novel point mutation in RpoB improves osmotolerance and succinic acid production in *Escherichia coli*. BMC Biotechnol 17:10. doi:10.1186/s12896-017-0337-6. 28193207 PMC5307762

[17] Silva IJ, Ortega AD, Viegas SC, García-Del Portillo F, Arraiano CM 2013 An RpoS-dependent sRNA regulates the expression of a chaperone involved in protein folding. RNA 19:1253–1265. doi:10.1261/rna.039537.113. 23893734 PMC3753932

[18] Timmermans J, van Melderen L 2009 Conditional essentiality of the *csrA* gene in *Escherichia coli*. J Bacteriol 191:1722–1724. doi:10.1128/JB.01573-08. 19103924 PMC2648183

[19] Holm AK, Blank LM, Oldiges M, Schmid A, Solem C, Jensen PR, Vemuri GN 2010 Metabolic and transcriptional response to cofactor perturbations in *Escherichia coli*. J Biol Chem 285:17498–17506. doi:10.1074/jbc.M109.095570. 20299454 PMC2878514

[20] Bak G, Han K, Kim D, Lee Y 2014 Roles of *rpoS*-activating small RNAs in pathways leading to acid resistance of *Escherichia coli*. Microbiologyopen 3:15–28. doi:10.1002/mbo3.143. 24319011 PMC3937726

[21] Rau MH, Bojanovic K, Nielsen AT, Long KS 2015 Differential expression of small RNAs under chemical stress and fed-batch fermentation in E. coli. BMC Genomics 16:1051. doi:10.1186/s12864-015-2231-8. 26653712 PMC4676190

[22] Klein G, Raina S 2017 Small regulatory bacterial RNAs regulating the envelope stress response. Biochm Soc Trans 45:417–425. doi:10.1042/BST20160367. PMC5736990 28408482

[23] Huisman GW, Kolter R 1994 Sensing starvation: a homoserine lactone-dependent signalling pathway in *Escherichia coli*. Science 265:537–539. doi:10.1126/science.7545940. 7545940

[24] Weikert C, Canonaco F, Sauer U, Bailey JE 2000 Co-overexpression of RspAB improves recombinant protein production in *Escherichia coli*. Metab Eng 2:293–299. doi:10.1006/mben.2000.0163. 11120641

[25] Han MJ, Park SJ, Park TJ, Lee SY 2004 Roles and applications of small heat shock proteins in the production of recombinant proteins in *Escherichia coli*. Biotechnol Bioeng 88:426–436. doi:10.1002/bit.20227. 15382106

[26] Price GP, St John AC 2000 Purification and analysis of expression of the stationary phase-inducible Slp lipoprotein in *Escherichia coli*: role of the Mar system. FEMS Microbiol Lett 193:51–56. doi:10.1111/j.1574-6968.2000.tb09401.x. 11094278

[27] Tucker DL, Tucker N, Conway T 2002 Gene expression profiling of the pH response in *Escherichia coli*. J Bacteriol 184:1651–1658. 10.1128/JB.184.23.6551-6558.2002 PMC135413 12426343

[28] Schliep M, Ryall B, Ferenci T 2012 The identification of global patterns and unique signatures of proteins across 14 environments using outer membrane proteomics of bacteria. Mol Biosyst 8:3017–3027. doi:10.1039/c2mb25212k. 22956018

[29] Yang YT, Bennett GN, San KY 2001 The effects of feed and intracellular pyruvate levels on the redistribution of metabolic fluxes in *Escherichia coli*. Metab Eng 3:115–123. doi:10.1006/mben.2000.0166. 11289788

[30] Bennett BD, Kimball EH, Gao M, Osterhout R, van Dien SJ, Rabinowitz JD 2009 Absolute metabolite concentrations and implied enzyme active site occupancy in *Escherichia coli*. Nat Chem Biol 5:593–599. doi:10.1038/nchembio.186. 19561621 PMC2754216

[31] Becker J, Wittmann C 2015 Advanced biotechnology: metabolically engineered cells for the bio-based production of chemicals and fuels, materials, health-care products. Angew Chem Int Ed Engl 54:3328–3350. doi:10.1002/anie.201409033. 25684732

[32] de Marco A, Deuerling E, Mogk A, Tomoyasu T, Bukau B 2007 Chaperone-based procedure to increase yields of soluble recombinant proteins produced in *E. coli*. BMC Biotechnol 7:32. doi:10.1186/1472-6750-7-32. 17565681 PMC1904446

[33] Cheng CH, Lee WC 2010 Protein solubility and differential proteomic profiling of recombinant *Escherichia coli* overexpressing double-tagged fusion proteins. Microb Cell Fact 9:63. doi:10.1186/1475-2859-9-63. 20799977 PMC2940792

[34] Wu H, Li ZM, Zhou L, Ye Q 2007 Improved succinic acid production in the anaerobic culture of an *Escherichia coli pflB ldhA* double mutant as a result of enhanced anaplerotic activities in the preceding aerobic culture. Appl Environ Microbiol 73:7837–7843. doi:10.1128/AEM.01546-07. 17951436 PMC2168152

[35] Baba T, Ara T, Hasegawa M, Takai Y, Okumura Y, Baba M, Datsenko KA, Tomita M, Wanner BL, Mori H 2006 Construction of *Escherichia coli* K-12 in-frame, single-gene knockout mutants: the Keio collection. Mol Syst Biol 2:2006.0008. doi:10.1038/msb4100050. PMC1681482 16738554

[36] Datsenko KA, Wanner BL 2000 One-step inactivation of chromosomal genes in *Escherichia coli* K-12 using PCR products. Proc Natl Acad Sci U S A 97:6640–6645. doi:10.1073/pnas.120163297. 10829079 PMC18686

[37] Ritchie ME, Silver J, Oshlack A, Holmes M, Diyagama D, Holloway A, Smyth GK 2007 A comparison of background correction methods for two-colour microarrays. Bioinformatics 23:2700–2707. doi:10.1093/bioinformatics/btm412. 17720982

[38] Smyth GK, Speed T 2003 Normalization of cDNA microarray data. Methods 31:265–273. doi:10.1016/S1046-2023(03)00155-5. 14597310

[39] Gentleman RC, Carey VJ, Bates DM, Bolstad B, Dettling M, Dudoit S, Ellis B, Gautier L, Ge Y, Gentry J, Hornik K, Hothorn T, Huber W, Iacus S, Irizarry R, Leisch F, Li C, Maechler M, Rossini AJ, Sawitzki G, Smith C, Smyth G, Tierney L, Yang JY, Zhang J 2004 Bioconductor: open software development for computational biology and bioinformatics. Genome Biol 5:R80. doi:10.1186/gb-2004-5-10-r80. 15461798 PMC545600

[40] Ritchie ME, Phipson B, Wu D, Hu Y, Law CW, Shi W, Smyth GK 2015 limma powers differential expression analyses for RNA-sequencing and microarray studies. Nucleic Acids Res 43:e47. doi:10.1093/nar/gkv007. 25605792 PMC4402510

[41] Phipson B, Lee S, Majewski IJ, Alexander WS, Smyth GK 2016 Robust hyperparameter estimation protects against hypervariable genes and improves power to detect differential expression. Ann Appl Stat 10:946. doi:10.1214/16-AOAS920. 28367255 PMC5373812

[42] Benjamini Y, Hochberg Y 1995 Controlling the false discovery rate: a practical and powerful approach to multiple testing. J R Stat Soc Series B Stat Methodol 57:289–300. doi:10.1111/j.2517-6161.1995.tb02031.x.

[43] Spura J, Reimer LC, Wieloch P, Schreiber K, Buchinger S, Schomburg D 2009 A method for enzyme quenching in microbial metabolome analysis successfully applied to Gram-positive and Gram-negative bacteria and yeast. Anal Biochem 394:192–201. doi:10.1016/j.ab.2009.07.016. 19615328

[44] Davey MP, Burrell MM, Woodward FI, Quick WP 2008 Population-specific metabolic phenotypes of *Arabidopsis lyrata* ssp. *petraea*. New Phytol 177:380–388. doi:10.1111/j.1469-8137.2007.02282.x. 18028292

[45] Walker H 2011 Metabolite profiling of plant tissues by electrospray mass spectrometry *In* De Bruijn FJ (ed), Handbook of microbial ecology. I. Metagenomics and complementary approaches. Wiley-Blackwell, Hoboken, NJ.

[46] Smith CA, Want EJ, O'Maille G, Abagyan R, Siuzdak G 2006 XCMS: processing mass spectrometry data for metabolite profiling using nonlinear peak alignment, matching, and identification. Anal Chem 78:779–787. doi:10.1021/ac051437y. 16448051

[47] Kazmi SA, Ghosh S, Shin DG, Hill DW, Grant DF 2006 Alignment of high resolution mass spectra: development of a heuristic approach for metabolomics. Metabolomics 2:75–83. doi:10.1007/s11306-006-0021-7.

[48] Overy S, Walker H, Malone S, Howard T, Baxter C, Sweetlove L, Hill SA, Quick WP 2004 Application of metabolite profiling to the identification of traits in a population of tomato introgression lines. J Exp Bot 56:287–296. doi:10.1093/jxb/eri070. 15596481

[49] Fischer R, Kessler BM 2015 Gel-aided sample preparation (GASP)—a simplified method for gel-assisted proteomic sample generation from protein extracts and intact cells. Proteomics 15:1224–1229. doi:10.1002/pmic.201400436. 25515006 PMC4409837

[50] Perez-Riverol Y, Csordas A, Bai J, Bernal-Llinares M, Hewapathirana S, Kundu DJ, Inuganti A, Griss J, Mayer G, Eisenacher M, Pérez E, Uszkoreit J, Pfeuffer J, Sachsenberg T, Yilmaz S, Tiwary S, Cox J, Audain E, Walzer M, Jarnuczak AF, Ternent T, Brazma A, Vizcaíno JA 2019 The PRIDE database and related tools and resources in 2019: improving support for quantification data. Nucleic Acids Res 47:D442–D450. doi:10.1093/nar/gky1106. 30395289 PMC6323896

[51] Tautenhahn R, Böttcher C, Neumann S 2008 Highly sensitive feature detection for high resolution LC/MS. BMC Bioinformatics 9:504. doi:10.1186/1471-2105-9-504. 19040729 PMC2639432

[52] Kuhl C, Tautenhahn R, Böttcher C, Larson TR, Neumann S 2012 CAMERA: an integrated strategy for compound spectra extraction and annotation of liquid chromatography/mass spectrometry data sets. Anal Chem 84:283–289. doi:10.1021/ac202450g. 22111785 PMC3658281

[53] Guha R 2007 Chemical informatics functionality in R. J Stat Softw 18:6.

[54] Guo AC, Jewison T, Wilson M, Liu Y, Knox C, Djoumbou Y, Lo P, Mandal R, Krishnamurthy R, Wishart DS 2012 ECMDB: the *E. coli* Metabolome Database. Nucleic Acids Res 41:D625–30. doi:10.1093/nar/gks992. 23109553 PMC3531117

[55] Kind T, Liu KH, Lee DY, DeFelice B, Meissen JK, Fiehn O 2013 LipidBlast *in silico* tandem mass spectrometry database for lipid identification. Nat Methods 10:755–758. doi:10.1038/nmeth.2551. 23817071 PMC3731409

[56] Koelmel JP, Kroeger NM, Ulmer CZ, Bowden JA, Patterson RE, Cochran JA, Beecher CWW, Garrett TJ, Yost RA 2017 LipidMatch: an automated workflow for rule-based lipid identification using untargeted high-resolution tandem mass spectrometry data. BMC Bioinformatics 18:331. doi:10.1186/s12859-017-1744-3. 28693421 PMC5504796

[57] Howell DM, Xu H, White RH 1999 (*R*)-Citramalic acid synthase in methanogenic archaea. J Bacteriol 181:331–333. 9864346 10.1128/jb.181.1.331-333.1999 PMC103565

[58] Schneider CA, Rasband WS, Eliceiri KW 2012 NIH Image to ImageJ: 25 years of image analysis. Nat Methods 9:671–675. 22930834 10.1038/nmeth.2089 PMC5554542

