# Supplementary figures and images for: Systems Analyses Reveal the Resilience of Escherichia coli Physiology during Accumulation and Export of the Nonnative Organic Acid Citramalate
Source: mSystems. 2019 Jun 11;4(4):e00187-19. doi: 10.1128/mSystems.00187-19 (PMC6561320; doi:10.1128/mSystems.00187-19)

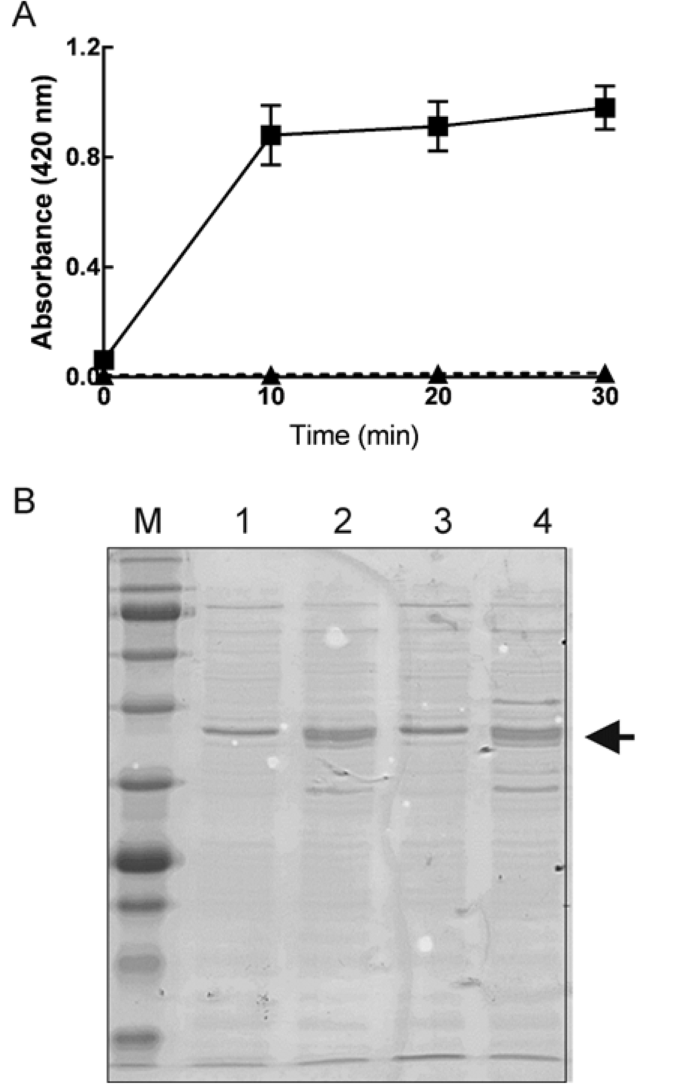

Supplement: FIG S1 [file mSystems.00187-19-sf001.tif]

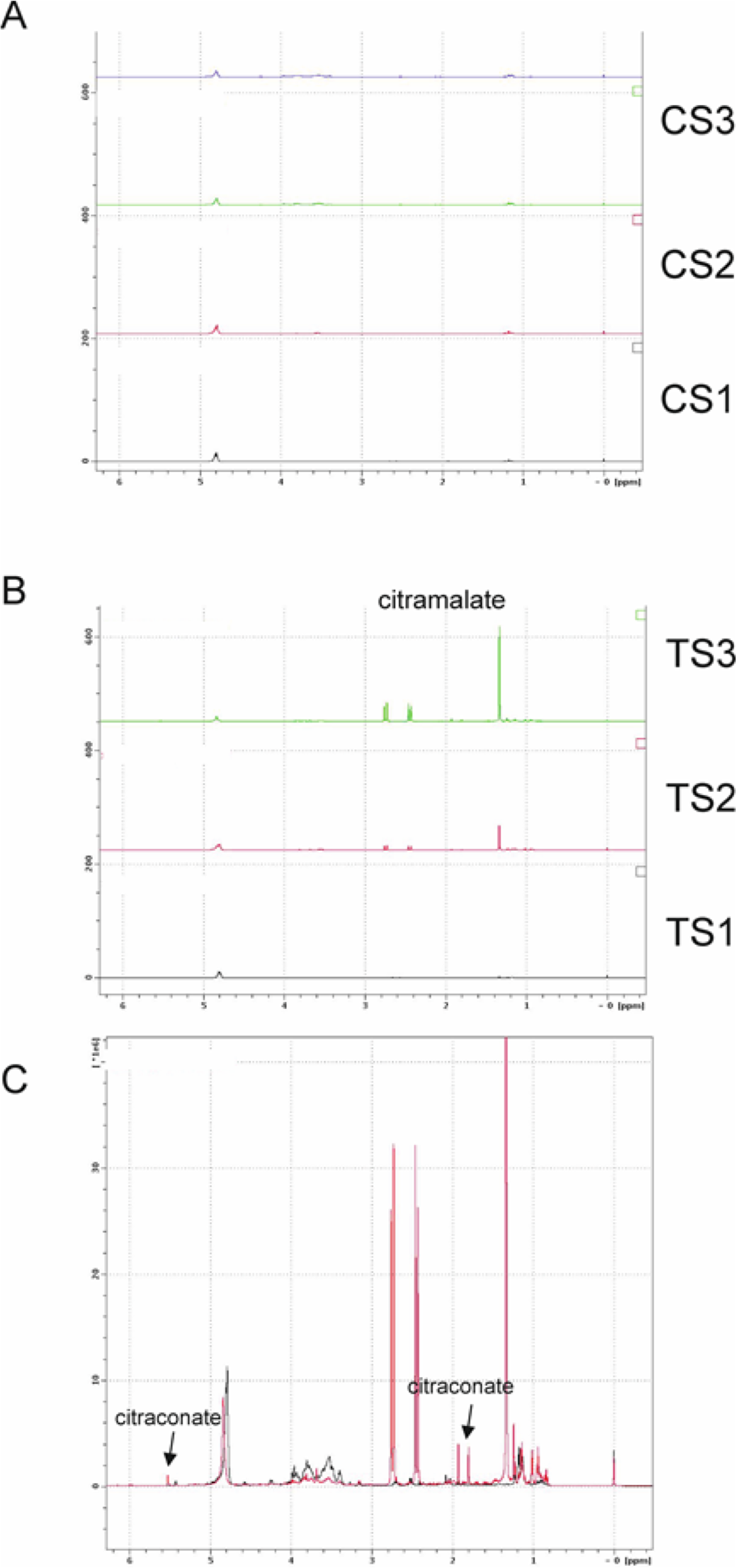

Supplement: FIG S2 [file mSystems.00187-19-sf002.tif]

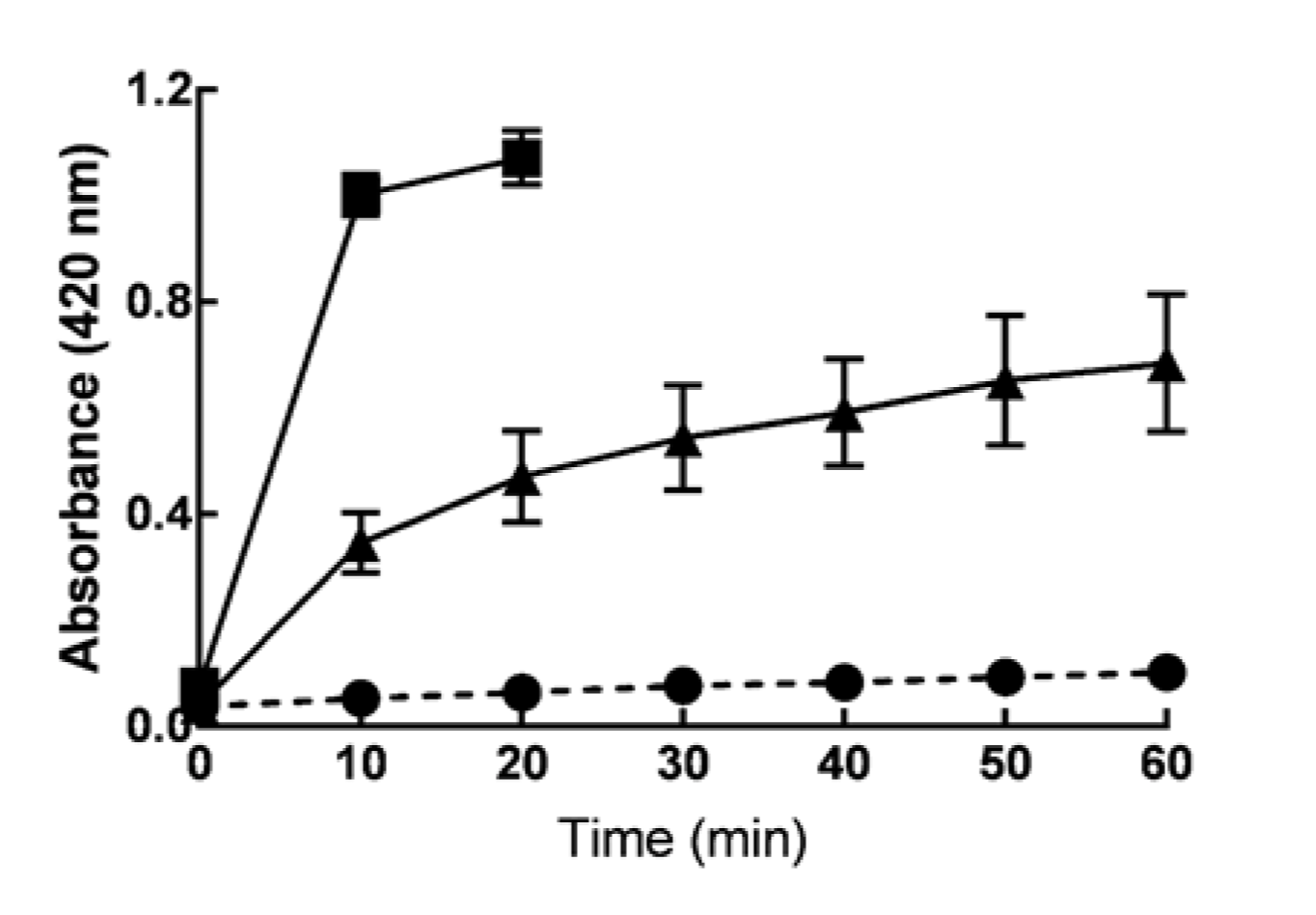

Supplement: FIG S3 [file mSystems.00187-19-sf003.tif]

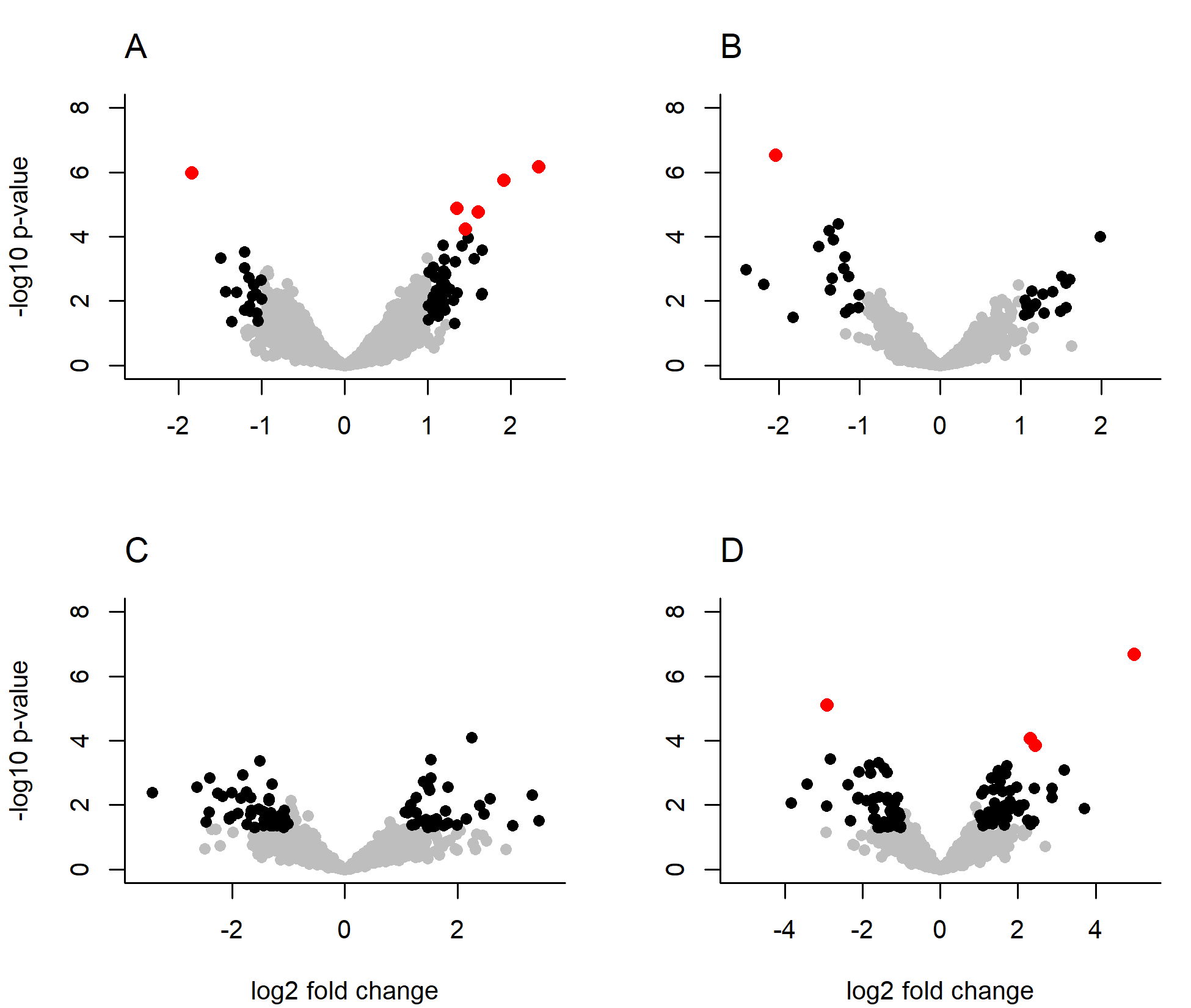

Supplement: FIG S4 [file mSystems.00187-19-sf004.tif]

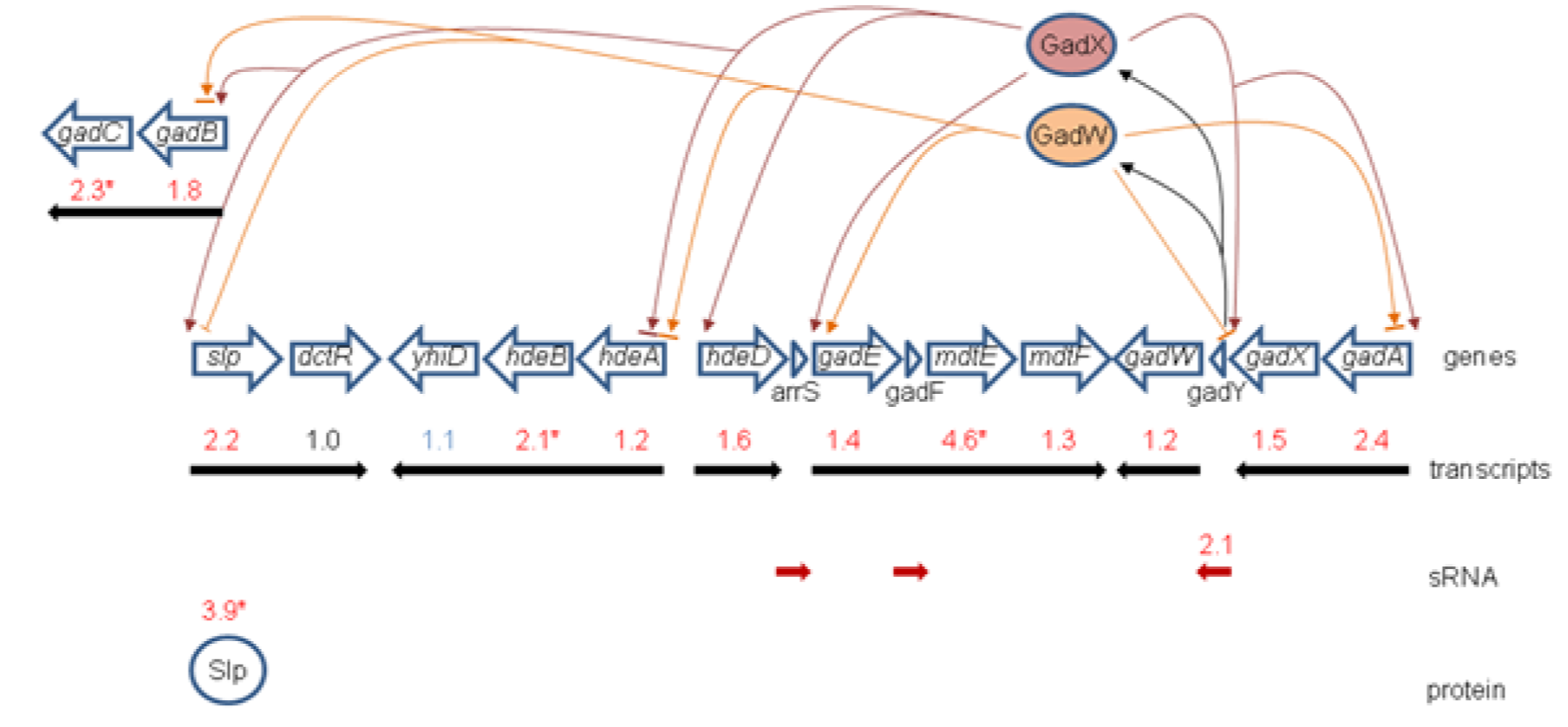

Supplement: FIG S5 [file mSystems.00187-19-sf005.tif]
