# Supplementary material for: Systems Analyses Reveal the Resilience of Escherichia coli Physiology during Accumulation and Export of the Nonnative Organic Acid Citramalate
Source: mSystems. 2019 Jun 11;4(4):e00187-19. doi: 10.1128/mSystems.00187-19 (PMC6561320; doi:10.1128/mSystems.00187-19)
Supplement: TABLE S4 [file mSystems.00187-19-st004.docx]

| \| **Gene(s) deleted** \| **Citramalate produced compared to wild type** \| **Glucose to citramalate conversion efficiency compared to wild type** \| \| \| --- \| --- \| --- \| --- \| \| *alaE* \| 107.2 ± 4.7% \| 97.2 ± 6.5% \| \| *mdtE* \| 104.8 ± 1.5% \| 106.4 ± 3.5% \| \| *nmpC* \| 89.6 ± 4.3% \| 83.1 ± 4.0% \| \| *slp* \| 109.5 ± 4.3% \| 102.5 ± 3.7% \| \| *acrA* \| 90.4 ± 4.6% \| 99.1 ± 11.7% \| \| *mscS* \| 102.9 ± 4.2% \| 106.1 ± 5.5% \| \| *mscL* \| 100.5 ± 6.7% \| 87.3 ± 6.9% \| \| *mscS, mscL* \| 107.4 ± 4.0% \| 111.91 ± 2.8% \| |
| --- | --- | --- | --- | --- | --- | --- | --- | --- | --- | --- | --- | --- | --- | --- | --- | --- | --- | --- | --- | --- | --- | --- | --- | --- | --- | --- | --- | --- |
|  |
